# Biocompatible glyconanomaterials based on HPMA-copolymer for specific targeting of galectin-3

**DOI:** 10.1186/s12951-018-0399-1

**Published:** 2018-09-20

**Authors:** P. Bojarová, M. R. Tavares, D. Laaf, L. Bumba, L. Petrásková, R. Konefał, M. Bláhová, H. Pelantová, L. Elling, T. Etrych, P. Chytil, V. Křen

**Affiliations:** 1 Institute of Microbiology, Czech Academy of Sciences, Vídeňská 1083, 14220 Prague 4, Czech Republic; 2 Institute of Macromolecular Chemistry, Czech Academy of Sciences, Heyrovský Sq. 2, 16206 Prague 6, Czech Republic; 3 Laboratory for Biomaterials, Institute for Biotechnology and Helmholtz-Institute for Biomedical Engineering, RWTH Aachen University, Pauwelsstraße 20, 52074 Aachen, Germany

**Keywords:** Carbohydrate, ELISA, Galectin-3, Glyconanomaterial, HPMA copolymer, Surface plasmon resonance

## Abstract

**Background:**

Galectin-3 (Gal-3) is a promising target in cancer therapy with a high therapeutic potential due to its abundant localization within the tumor tissue and its involvement in tumor development and proliferation. Potential clinical application of Gal-3-targeted inhibitors is often complicated by their insufficient selectivity or low biocompatibility. Nanomaterials based on *N*-(2-hydroxypropyl)methacrylamide (HPMA) nanocarrier are attractive for in vivo application due to their good water solubility and lack of toxicity and immunogenicity. Their conjugation with tailored carbohydrate ligands can yield specific glyconanomaterials applicable for targeting biomedicinally relevant lectins like Gal-3.

**Results:**

In the present study we describe the synthesis and the structure-affinity relationship study of novel Gal-3-targeted glyconanomaterials, based on hydrophilic HPMA nanocarriers. HPMA nanocarriers decorated with varying amounts of Gal-3 specific epitope GalNAcβ1,4GlcNAc (LacdiNAc) were analyzed in a competitive ELISA-type assay and their binding kinetics was described by surface plasmon resonance. We showed the impact of various linker types and epitope distribution on the binding affinity to Gal-3. The synthesis of specific functionalized LacdiNAc epitopes was accomplished under the catalysis by mutant β-*N*-acetylhexosaminidases. The glycans were conjugated to statistic HPMA copolymer precursors through diverse linkers in a defined pattern and density using Cu(I)-catalyzed azide–alkyne cycloaddition. The resulting water-soluble and structurally flexible synthetic glyconanomaterials exhibited affinity to Gal-3 in low μM range.

**Conclusions:**

The results of this study reveal the relation between the linker structure, glycan distribution and the affinity of the glycopolymer nanomaterial to Gal-3. They pave the way to specific biomedicinal glyconanomaterials that target Gal-3 as a therapeutic goal in cancerogenesis and other disorders.

**Electronic supplementary material:**

The online version of this article (10.1186/s12951-018-0399-1) contains supplementary material, which is available to authorized users.

## Background

Most transmembrane proteins in vivo carry an information-rich glycan pattern that mediates major physiological and pathological processes, such as cellular adhesion, inter-cell exchange of information, defense mechanisms, immune response [1] as well as, e.g., cancerogenesis [2]. The so-called “sugar-code” is deciphered by lectins, abundant glycan-binding proteins. Thanks to the natural presentation of glycans in a multivalent fashion, the relatively weak monovalent lectin-glycan interaction is amplified by the “cluster glycoside effect”, and, as a result, the system exhibits a cumulative affinity (i.e. avidity), which is higher than the sum of affinity contributions of individual binding events [3].

The described avidity pattern can be mimicked by synthetic multivalent glycoconjugates. They are based on a range of carrier scaffolds [4], of which glycopolymers embrace an exceptional structural and functional variability [5, 6]. The synthesis of glycopolymers may advantageously employ Cu(I)-catalyzed azide–alkyne cycloaddition (CuAAC)—a type of “click chemistry” [7] that couples unprotected glycans under the formation of a triazole moiety.

The synthetic nanocarrier of *N*-(2-hydroxypropyl)methacrylamide (HPMA) copolymer is especially attractive for the construction of in vivo applicable nanomaterials since it is fully biocompatible, water soluble and lacks any toxicity or immunogenicity [8]. Furthermore, therapeutics or drugs conjugated to HPMA-based nanocarriers exhibit considerably reduced and/or decelerated dose-related negative side effects on healthy organs, with an enhanced therapeutic outcome [9]. The administration of a polymer-conjugated drug (the “polymer prodrug”) results in a favorable pharmacokinetics [10, 11], which enables prolonged circulation in the body and increased uptake in solid tumors or inflammation sites [12]. The polymer nanocarriers are passively accumulated in solid tumors thanks to the enhanced permeability and retention (EPR) effect [13] and similarly in inflammation sites through the inflammatory cell-mediated sequestration (ELVIS) [12]. Additional active targeting of polymer prodrugs through suitable epitopes specific for tumor or inflammation issue, such as in the case of carbohydrate ligands binding lectins, brings a desired increase in the selectivity and efficacy of targeted interaction.

A promising target for directing glycopolymers to cancer-stricken tissue is galectin-3 (Gal-3)—the only representative of chimeric galectins in mammals [14]. Gal-3 is overexpressed in many cancer types, mainly but not exclusively in those related to the gastrointestinal tract. It takes part in neoplasia and metastasis, tumorigenesis, angiogenesis, as well as immune escape of tumor cells [15]. The Gal-3 molecule comprises a conserved C-terminal carbohydrate recognition domain (CRD) with the binding site for up to a tetrasaccharide ligand, and an N-terminal domain responsible for Gal-3 cross-linking and oligomerization [16]. Gal-3 binds β-galactoside-terminated glycans like all galectins; however, we have recently discovered a selective carbohydrate epitope for Gal-3, *N*,*N*´-diacetyllactosamine (GalNAcβ1,4GlcNAc, LacdiNAc), which is not recognized by the similarly frequent galectin-1 [17, 18]. In mammals, this epitope is rarely present in some *N*-and *O*-linked glycoproteins [19] and it also occurs in several cancer types [16–20].

Gal-3 is a notoriously difficult molecule for evoking multivalence effect since it is present as a monomer in solution [21] and, though it is known to oligomerize in the presence of multivalent ligands by several mechanisms [22–24], this process is still rather unclear. Multivalent presentation of disaccharide ligands with no (carbohydrate or other) extension at the non-reducing end [16–18] leads to an interaction only with the conserved part of the Gal-3 binding pocket [25]. Therefore, conjugated disaccharides are less efficient ligands of Gal-3 than more complex oligosaccharide glycans [26–28]. On the other hand, the complex glycans, though more efficient in Gal-3 binding, are difficult and expensive to routinely synthesize in hundreds-of-milligram-amounts needed for larger-scale testing.

In the past 15 years, Gal-3 has been targeted by various low-molecular inhibitors [25] as well as by multivalent glycoconjugates based on miscellaneous scaffolds [29, 30]. Some scarce information has been published on HPMA copolymers carrying simple commercially available sugars (galactose, lactose) to act as ligands of Gal-3 [31]. Though current knowledge encompasses dozens of types of Gal-3 inhibitors, just a handful of them has ever reached beyond the doors of academic research laboratories [32]. The major reasons comprise insufficient selectivity for Gal-3, a difficult scalability of the synthetic process, especially when using complex oligosaccharide ligands, and/or low biocompatibility of the carrier scaffold.

The highly biocompatible glycan-decorated HPMA copolymers described in this work are predestined for in vivo application. In this work we present an efficient chemoenzymatic synthesis [33] of the selective LacdiNAc disaccharide epitope bearing various functionalities using the Tyr470His mutant of the β-*N*-acetylhexosaminidase from *Talaromyces flavus* developed in our laboratory [34]. We have thoroughly analyzed the impact of structural organization and the type of conjugation of LacdiNAc disaccharide on the binding of the HPMA glycopolymers to Gal-3. We present a structure-affinity relationship study on 16 glycopolymers containing 3–29 mol. % of LacdiNAc epitope attached with four different spacers via two different carbohydrate functionalities. The affinity to Gal-3 was determined in a competitive ELISA assay and its kinetics was analyzed by surface plasmon resonance (SPR). The assembled results shed light on the structure-affinity relationship of these attractive glyconanomaterials as promising candidates for biomedical applications targeting Gal-3 for diagnosis or therapy.

## Methods

### General

TLC was performed using aluminium sheets with Silica Gel 60 precoating (F254 Merck, D); the spots were visualized by UV light (254 nm), then sprayed with 5% H_2_SO_4_ in ethanol and charred. Asialofetuin was purchased from GlycoTech (Gaithersburg, MD, USA). Lactose (Galβ1,4Glc) was bought from Lachema (CZ) and LacNAc (Galβ1,4GlcNAc) from Carbosynth Ltd. (UK). *p*-Nitrophenyl 2-acetamido-2-deoxy-β-d-galactopyranoside (*p*NP-GalNAc) and *p*-nitrophenyl 2-acetamido-2-deoxy-β-d-glucopyranoside (*p*NP-GlcNAc) were obtained from Gold Biotechnology (MO, USA). *N*-(3-*tert*-butoxycarbonyl-aminopropyl)methacrylamide (APMA-Boc) was purchased from Polysciences, Inc. (PA, USA) and dibenzocyclooctyne (DBCO) amine from Click Chemistry Tools (AZ, USA). (4-Ethynylphenyl)methanamine hydrochloride and 3,5-diethynylbenzoic acid were obtained from Ark Pharm (IL, USA). Bovine serum albumin (BSA), 2-cyanopropan-2-yl dithiobenzoate (CTA), 4-cyano-4-(thiobenzoylthio)pentanoic acid, 2,2′-azobisisobutyronitrile (AIBN), 4,4′-azobis(4-cyanopentanoic acid) (ACVA), *N*,*N*-dimethylacetamide (DMA), *N*,*N*-dimethylformamide (DMF), dimethyl sulfoxide (DMSO), *N*,*N*-diisopropylethylamine (DIPEA), 4-dimethylaminopyridine, 1-ethyl-3-(3-dimethylaminopropyl)carbodiimide hydrochloride (EDC.HCl), 8-quinolinol, trifluoroacetic acid (TFA), 2-thiazoline-2-thiol and other chemicals and materials, if not stated otherwise, were purchased from Sigma.

### MS measurements

Mass spectra were measured on an LTQ Orbitrap XL hybrid mass spectrometer (Thermo Fisher Scientific, Waltham, USA) containing an electrospray ion source. The samples were injected via a 2 μL loop using methanol/water (4/1, v/v) mobile phase at a flow rate of 30 μL/min. For the internal calibration of the mass spectra of negatively charged ions, deprotonated palmitic acid was used as a lock mass. The Xcalibur software (Thermo Fisher Scientific) was used for processing the data.

### NMR measurements

NMR spectra of compounds **3**–**7** were acquired on a Bruker AVANCE III 600 and 700 MHz spectrometer (Bruker BioSpin, Rheinstetten, Germany) in D_2_O (99.98% D, ARMAR Chemicals, Dottingen, Switzerland) at 25 and 30 °C. Proton spectra were referenced to the residual signal of water (*δ*_H_ = 4.508 ppm); the signal of acetone (*δ*_C_ = 30.50 ppm) served as the reference for carbon chemical shifts. The set of NMR experiments (COSY, HSQC, HMBC, and 1d-TOCSY) were measured using Bruker software TopSpin 3.5. The resolution in ^1^H NMR spectra was enhanced by two-parameter double-exponential Lorentz–Gauss function applied before Fourier transformation; ^13^C signal-to-noise ratio was improved by line broadening (1 Hz). Glucose and galactose units were distinguished using extracted coupling constants; their anomeric configuration was confirmed by the magnitude of *J*_(H-1,H-2)_. *N*-Acetyl attachment was indicated by up-field shifted carbons C-2. Glycosidic linkage (1 → 4) was proved by the heteronuclear correlation of involved carbon C-4 with the corresponding anomeric proton.

The structures of polymer conjugates and their precursors were analyzed using NMR Bruker Avance III 600 spectrometer operating at 600.2 MHz using DMSO-*d*_6_ or D_2_O solvents. For the calculations, the integral intensity of signals of *δ* (ppm) = 3.67 (1 H, C*H*–OH) or *δ* (ppm) = 4.71 (1 H, CH–O*H*) of the HPMA monomer unit were used. The content of propargyl groups in copolymers **13**–**15** was determined using the integral intensity of signals of *δ* (ppm) = 8.36 (1 H, N*H*–CH_2_) and *δ* (ppm) = 3.85 (2 H, NH–C*H*_2_) of the propargylamide motif. In the case of polymer precursors **17** and **18** containing the ethynylphenyl linker, the integral intensity of signals of *δ* (ppm) = 8.47 (1 H, N*H*–CH_2_), *δ* (ppm) = 4.27 (2 H, NH–C*H*_2_ next to phenyl) and *δ* (ppm) = 4.13 (1 H, C*H* of triple bond) were employed. For calculating the content of diethynylphenyl linker in the polymer precursor **23**, we utilized integral intensity of signals of *δ* (ppm) = 8.70 (1 H, N*H*), δ (ppm) = 7.95 (2 H, C*H* of the phenyl), *δ* (ppm) = 7.71 (1 H, C*H* of the phenyl) and *δ* (ppm) = 4.37 (1 H, C*H* of triple bond). The content of carbohydrate in the polymer conjugates **24**–**32** and **35**–**39** was calculated using signals of *δ* (ppm) ≈ 7.98 (1 H, C*H* of triazole). In addition, we used signals of *δ* (ppm) ≈ 5.80 (1 H, C*H* of carbohydrate) for conjugates **36**, **37**, and **39**. In the case of conjugates **33** and **34**, the integral intensity of signals of *δ* (ppm) ≈ 1.95 (3 H, C*H*_3_ of GalNAc acetyl) was used.

### HPLC analyses

The course of transglycosylation reactions and the purity of prepared carbohydrates were monitored by high performance liquid chromatography (HPLC). Analyses were performed using a Shimadzu Prominence LC analytical system comprising a Shimadzu LC-20AD binary HPLC pump, a Shimadzu CTO-10AS column oven, a Shimadzu CBM-20A system controller, a Shimadzu SIL-20ACHT cooling autosampler, and a Shimadzu SPD-20MA diode array detector (Shimadzu, JP). The sample was dissolved in acetonitrile/water (4/1, v/v) and analyzed by a Prontosil 120-5-amino H column (250 × 4.6 mm, Bischoff chromatography, DE). Isocratic elution was used with 76% acetonitrile in water, v/v, as a mobile phase. The flow rate was 1 mL/min at 28 °C and the injection volume was 1 μL; the samples were detected at 200 nm. Retention times were as follows: *p*-nitrophenyl 2-acetamido-2-deoxy-β-d-galactopyranoside (**1**), 4.19 min; 2-acetamido-2-deoxy-d-glucopyranose (**2**), 7.42 min; 2-azidoethyl 2-acetamido-2-deoxy-β-d-glucopyranoside (**3**), 4.87 min; 2-acetamido-2-deoxy-β-d-glucopyranosyl azide (**4**), 5.01 min; 2-acetamido-2-deoxy-β-d-galactopyranosyl-(1 → 4)-2-acetamido-2-deoxy-d-glucopyranose (LacdiNAc; **5**), 6.57 min; 2-azidoethyl 2-acetamido-2-deoxy-β-d-galactopyranosyl-(1 → 4)-2-acetamido-2-deoxy-β-d-glucopyranoside (**6**), 6.55 min; 2-acetamido-2-deoxy-β-d-galactopyranosyl-(1 → 4)-2-acetamido-2-deoxy-β-d-glucopyranosyl azide (**7**), 6.70 min; 2-acetamido-2-deoxy-β-d-glucopyranosyl-(1 → 4)-2-acetamido-2-deoxy-β-d-glucopyranosyl azide, 7.11 min. Respective chromatograms are shown in Additional file 1: Figures S8–S11.

The purity of monomers *N*-(2-hydroxypropyl) methacrylamide (**8**; HPMA) and 3-(3-methacrylamidopropanoyl) thiazolidine-2-thione (**9**; MA-AP-TT) for polymer synthesis was assayed by a Shimadzu HPLC system equipped with a C18 reversed-phase Chromolith Performance RP-18e column (4.6 × 100 mm, Merck Millipore) and a diode array detector (Shimadzu SPD-M20A) using a mobile phase of water/acetonitrile/0.1% TFA with a gradient of 5–95% v/v acetonitrile at a flow rate of 5 mL/min. The molecular weights and dispersities were determined by a Shimadzu HPLC system equipped with gel permeation chromatography (GPC) columns (TSKgel Super SW3000, 300 × 4.6 mm; 4 µm or Superose 12 HR 10/300 GL), using refractive index Optilab-rEX (RI) detector and multiangle light scattering (MALS) detector (DAWN HELEOS II, Wyatt Technology Co., USA). Methanol/0.3 M sodium acetate buffer, pH 6.5 (4/1, v/v) at a flow rate of 0.3 mL/min was used as a mobile phase for the TSKgel column and 0.3 M sodium acetate buffer, pH 6.5 at a flow rate of 0.5 mL/minwas used for the Superose column.

### UV–Vis spectrophotometry

The content of the thiazolidine-2-thione (TT) and dibenzylcyclooctyne (DBCO) groups in the polymer precursors **10–12** and **16**, respectively, was determined by UV–Vis spectrophotometry in methanol using the molar absorption coefficients *ε*(TT) = 8400 L/mol/cm (*λ*_max_ = 305 nm) estimated for 3,3′-disulfanediylbis[1-(2-thioxothiazolidin-3-yl)propan-1-one] and *ε*(DBCO) = 13,000 L/mol/cm (*λ*_max_ = 292 nm) estimated for DBCO-amine. The content of amino groups in the polymer precursor **20** was determined by a modified 2,4,6-trinitrobenzene-1-sulfonic acid assay as described before [35].

### Dynamic light scattering

The hydrodynamic radii (*R*_H_) of copolymers in water (5 mg/mL) were measured using a Nano-ZS instrument (ZEN3600, Malvern). The intensity of the scattered light was detected at an angle *θ* = 173° using laser of a wavelength of 632.8 nm. To evaluate the dynamic light scattering data, the DTS(Nano) program was used. The values were a mean of at least five independent measurements and they were not extrapolated to zero concentration. Afterwards, the mixtures of copolymers with BSA (in a final concentration of 50 mg/mL which is comparable to the level usually present in human blood) were incubated for 1 h at r.t. and then they were evaluated by the same procedure.

### Protein production

#### Recombinant human His_6_-tagged galectin-3 (Gal-3)

The production and purification of Gal-3 for ELISA assays was basically done as described before [28, 36]. Briefly, Gal-3 was recombinantly expressed in *E. coli* Rosetta 2 (DE3) pLysS cells. The pre-cultures were cultivated (220 rpm, 37 °C, 60 mL in 0.5 L baffled flasks) in Lysogeny broth medium (LB; yeast extract 0.5, tryptone 1.0, NaCl 0.5% w/v, pH 7.4) containing ampicillin (100 mg/L) and chloramphenicol (34 mg/L) overnight. After 16 h, the 600-mL main cultures in Terrific broth medium (TB: yeast extract 2.4, tryptone 1.2% w/v, glycerin 0.4% v/v, KH_2_PO_4_ 17 mM, K_2_HPO_4_ 72 mM, pH 7.0) in 3 L baffled flasks containing antibiotics as above were inoculated with pre-cultures (1/10) and incubated at 37 °C and 150 rpm until an optical density (OD_600_) of 0.5–0.8. Then, isopropyl 1-thio-β-d-galactopyranoside (IPTG, 0.5 mM) was added to induce protein expression. After 24 h post-induction, the cells were harvested by centrifugation (5000 rpm, 20 min, 4 °C). For galectin purification, bacteria were suspended in cold equilibration buffer (20 mM Na_2_HPO_4_, 500 mM NaCl, 20 mM imidazole, pH 7.4) and sonicated on ice (two cycles per 30 s, 52% amplitude). Cell debris were removed by centrifugation (13,400 rpm, 15 min, 4 °C), the supernatant was filtered through 0.8 µm syringe filter and applied on HisTrap™ HP 5 mL column (GE Healthcare) according to manufacturer`s instructions in equilibration buffer. Gal-3 was eluted using elution buffer (20 mM Na_2_HPO_4_, 500 mM NaCl, 500 mM imidazole, pH 7.4). The Gal-3-containing fractions were combined and Gal-3 was dialyzed overnight against EPBS buffer (50 mM NaH_2_PO_4_, 150 mM NaCl, 2 mM ethylenediaminetetraacetic acid, pH 7.5) using SnakeSkin™ Dialysis Tubing (10 kDa MWCO, ThermoFisher Scientific). A usual yield was ca 12 g of cells per 1 L medium and 1.9 mg of pure Gal-3 per 1 g cells.

#### Biotinylated His_6_-tagged Gal-3 containing the AviTag sequence (GLNDIFEAQKIEWHE) (Gal-3-AVI)

The title construct Gal-3-AVI, used for SPR measurements, was prepared as described earlier [16]. It was expressed in *E. coli* BL21 (λDE3) cells along with an IPTG inducible plasmid carrying the *birA* gene. The cells were grown in mineral M9 medium supplemented with glycerol (20 g/L), yeast extract (20 g/L), ampicillin (150 mg/L) and chloramphenicol (10 mg/L) to an optical density (OD_600_) of 0.6, induced with IPTG (1 mM) in the presence of 50 μM d-biotin (Sigma), and grown for 4 h at 37 °C. The in vivo biotinylated His_6_-tagged Gal-3 protein was purified as described above. The Gal-3 concentrations were determined by Bradford assay calibrated with BSA. The molecular weight of Gal-3 and Gal-3-AVI was calculated from the amino acid sequence to be 28,023 and 30,550 Da, respectively.

#### Tyr470Phe and Tyr470His mutants of the β-*N*-acetylhexosaminidase from *Talaromyces flavus*

The title enzymes were basically prepared as described previously [34]. In short, the mutant variants were constructed by site-directed mutagenesis and they were extracellularly expressed in *Pichia pastoris* KM71H (Invitrogen, USA) [37]. The cells were cultivated at 28 °C and 220 rpm under the induction by methanol as recommended by manufacturer (EasySelect Pichia Expression Kit, Invitrogen, USA). The enzymes were purified in a single-step by cation-exchange chromatography on Fractogel EMD SO_3_^−^ (Merck, Germany) in 10 mM sodium citrate/phosphate buffer pH 3.5 with a linear gradient of 0-1 M NaCl using Äkta Purifier protein chromatography system (Amersham Biosciences, SW). The production yield reached ca. 10-20 mg of enzyme per 100 mL of culture medium. The enzymatic activity was determined in an end-point assay using 2 mM starting concentration of *p*NP-GlcNAc or *p*NP-GalNAc (**1**) in McIlvaine buffer pH 5.0 at 35 °C and 850 rpm with 0.1–0.25 U/mL of the respective β-*N*-acetylhexosaminidase. After 10 min, the reaction was stopped by adding 0.1 M Na_2_CO_3_ and the liberated *p*-nitrophenol was determined spectrophotometrically at 420 nm. One unit of enzymatic activity was defined as the amount of enzyme releasing 1 μmol of *p*-nitrophenol per minute under the described conditions.

### Synthetic procedures

#### 2-Azidoethyl 2-acetamido-2-deoxy-β-d-glucopyranoside (3) and 2-acetamido-2-deoxy-β-D-glucopyranosyl azide (**4**)

The title compounds **3** and **4** were prepared from GlcNAc (**2**) based on the procedures described previously [38, 39]. The ^1^H and ^13^C NMR data were consistent with the structure.

#### 2-Acetamido-2-deoxy-β-d-galactopyranosyl-(1 → 4)-2-acetamido-2-deoxy-d-glucopyranose (LacdiNAc; **5**)

*p*NP-GalNAc (**1**; 41 mg, 0.12 mmol), GlcNAc (**2**; 106 mg, 0.48 mmol), and the Tyr470His mutant of the β-*N*-acetylhexosaminidase from *T. flavus* (1.4 U, 13.4 mg, 480 μL) were incubated in a mixture of acetonitrile 10% v/v) in McIlvaine buffer pH 5.0 (total reaction volume 2.4 mL) at 45 °C and 1000 rpm. The reaction was monitored by HPLC and TLC (propan-2-ol/H_2_O/NH_4_OH aq., 7/2/1, v/v/v). After 2 h, the conversion of *p*NP-GalNAc was almost complete and another batch of *p*NP-GalNAc was added (18 mg, 0.05 mmol). After 4 h, the reaction was stopped by boiling for 2 min, and, upon cooling down to r.t. (room temperature), the mixture was centrifuged (13,500 rpm; 10 min) and loaded onto a Biogel P-2 column (2.6 × 100 cm, Bio-Rad, USA) using water as a mobile phase at an elution rate of 8 mL/h. The fractions containing product were combined and lyophilized; the title compound **5** was obtained as a white solid (40 mg, 0.094 mmol, 58% isolated yield). HRMS (ESI^+^): found *m*/*z* 447.15845 (expected 447.15853 for [M + Na]^+^, C_16_H_28_O_11_N_2_Na). For the respective NMR and MS data, see Additional file 1: Table S1 and Figures S1, S5.

#### Enzymatic synthesis of disaccharides **6** and **7**

To monitor the reaction progress at an analytical scale, *p*NP-GalNAc donor (**1**; 50 mM) and 2-azidoethyl 2-acetamido-2-deoxy-β-d-glucopyranoside acceptor (**3**; 50–250 mM) or 2-acetamido-2-deoxy-β-d-glucopyranosyl azide acceptor (**4**; 50–250 mM) were suspended in a mixture of acetonitrile (0–30% v/v) in 50 mM citrate–phosphate buffer (pH 5.0), and the Tyr470His mutant of the β-*N*-acetylhexosaminidase from *T. flavus* (0.5–5.8 mg/mL, 0.1–1.7 U/mL) was added. The reaction mixtures were incubated at 35–45 °C under shaking (1000 rpm) for up to 72 h. Aliquots (10 μL) were taken at regular time intervals and analyzed by HPLC. In parallel, the progress of the reaction was monitored by TLC (propan-2-ol/H_2_O/NH_4_OH aq., 7/2/1, v/v/v).

#### 2-Azidoethyl 2-acetamido-2-deoxy-β-d-galactopyranosyl-(1 → 4)-2-acetamido-2-deoxy-β-d-glucopyranoside (**6**)

*p*NP-GalNAc (**1**; 41 mg, 0.12 mmol), acceptor **3** (104 mg, 0.36 mmol), and the Tyr470His mutant of the β-*N*-acetylhexosaminidase from *T. flavus* (1.4 U, 13.4 mg, 480 μL) were incubated in a mixture of acetonitrile (10% v/v) in McIlvaine buffer pH 5.0 (total reaction volume 2.4 mL) at 45 °C and 1000 rpm and monitored by HPLC and TLC. After 5 h, the reaction was stopped by boiling for 2 min, and, upon cooling down to r.t., the mixture was centrifuged (13,500 rpm; 10 min and loaded onto a Biogel P-2 column (2.6 × 100 cm, Bio-Rad, USA) using water as a mobile phase at the elution rate of 8 mL/h. Pure acceptor **3** was partially recovered (39 mg). The fractions containing the product were combined and lyophilized; the title compound **6** was obtained as a white solid (29 mg, 0.058 mmol, 48% isolated yield). HRMS (ESI^−^): found *m*/*z* 492.19437 (expected 492.19473 for [M−H]^−^, C_18_H_30_O_11_N_5_). For the respective NMR data, see Additional file 1: Table S2 and Figures S2, S6.

#### 2-Acetamido-2-deoxy-β-d-galactopyranosyl-(1 → 4)-2-acetamido-2-deoxy-β-d-glucopyranosyl azide (**7**)

*p*NP-GalNAc (**1**; 41 mg, 0.12 mmol), acceptor **4** (118 mg, 0.48 mmol), and the Tyr470His mutant of the β-*N*-acetylhexosaminidase from *T. flavus* (1.4 U, 12.6 mg, 450 μL) were incubated in a mixture of acetonitrile (10% v/v) in McIlvaine buffer pH 5.0 (total reaction volume 2.4 mL) at 45 °C and 1000 rpm and monitored by HPLC and TLC. After 6.5 h, the reaction was stopped by boiling for 2 min, and, upon cooling down to r.t., the mixture was centrifuged (13,500 rpm; 10 min) and loaded onto a Biogel P-2 column (2.6 × 100 cm, Bio-Rad, USA) using water as a mobile phase at the elution rate of 8 mL/h. Pure acceptor **4** was partially recovered (34 mg). The fractions containing the product were combined and lyophilized; the title compound **5** was obtained as a white solid (19 mg, 0.042 mmol, 35% isolated yield). HRMS (ESI^−^): found *m*/*z* 448.16819 (expected 448.16852 for [M−H]^−^, C_16_H_26_N_5_O_10_). For the respective NMR and MS data, see Additional file 1: Table S3, Figures S3, S7.

#### Copolymers **13**–**18** and **23**

The monomers HPMA (**8**) and MA-AP-TT (**9**) were prepared as described previously [40, 41]. The polymer precursors poly(HPMA-*co*-MA-AP-TT) (**10**–**12**) were prepared by reversible addition–fragmentation chain transfer (RAFT) polymerization of **8** and **9** using the chain transfer agent 2-cyanopropan-2-yl dithiobenzoate (CTA) and the initiator 2,2′-azobisisobutyronitrile (AIBN), followed by dithiobenzoate end group removal. The reaction conditions were adopted from our previous work [39]. The molar ratio of monomer/CTA/AIBN was 350/2/1 (**10**, **11**) or 450/2/1 for (**12**) and the molar ratio of monomers HPMA/MA-AP-TT was 9/1 (**10**), 8/2 (**11**) or 7/3 (**12**). The copolymers poly(HPMA-*co*-MA-AP-propargyl) (**13**–**15**), poly(HPMA-*co*-MA-AP-DBCO) (**16**), and poly(HPMA-*co*-MA-AP-ethynylphenyl) (**17**, **18**) were synthesized via aminolysis of thiazolidine-2-thione groups of the polymer precursors poly(HPMA-*co*-MA-AP-TT) (**10**–**12**) with the amine-functionalized spacers propargylamine, DBCO-amine, and (4-ethynylphenyl)methanamine, respectively, in DMA using DIPEA as a base at r.t. The polymer precursor poly(HPMA-*co*-APMA) (**20**) was prepared by RAFT polymerization of HPMA (**8**) and APMA-Boc (**19**), using the chain transfer agent 4-cyano-4-(thiobenzoylthio)pentanoic acid and the initiator 4,4′-azobis(4-cyanopentanoic acid) (ACVA), followed by the removal of dithiobenzoate end group as described above and the deprotection of amine group using TFA. The copolymer poly(HPMA-*co*-APMA-diethynylphenyl) (**23**) was prepared by aminolytic reaction of **21** and 3,5-diethynylbenzoyl thiazolidine-2-thione (**22**) by the same procedure as polymer precursors **13**-**18**. Compound **22** was prepared by the reaction of 2-thiazoline-2-thiol and 3,5-diethynylbenzoic acid (**21**) using carbodiimide. The detailed synthetic procedures are described in Additional file 1: Section 2.1. Representative NMR data are given in Additional file 1: Figures S12–S16.

#### Synthesis of glyconanomaterials **24**–**39**

Glyconanomaterials **24**–**32** and **35**–**39** were prepared by the reaction of triple bonds in propargyl, ethynylphenyl or diethynylphenyl spacers of polymer precursors with the respective azido-functionalized carbohydrates **6** or **7** by CuAAC as described previously [39]. The reaction time was 1 h for the reactions employing a propargyl linker (**24**–**32**), and 20 h for the reactions involving a phenyl linker (**35**–**39**). The glyconanomaterials **33** and **34** were prepared by the reaction of DBCO groups of polymer precursor **16** and the respective azido-functionalized carbohydrates **6** or **7** by the copper-free azide–alkyne cycloaddition in methanol. Detailed synthetic procedures are described in Additional file 1: Section 2.2. Representative NMR data are given in Additional file 1: Figures. S17–S24.

#### Competitive ELISA assay with Gal-3

The capability of glyconanomaterials **24**–**39** to inhibit binding of Gal-3 to the immobilized asialofetuin (ASF, Sigma Aldrich, Steinheim, Germany) was determined in the competitive ELISA design. The F16 Maxisorp NUNC-Immuno Modules (Thermo Scientific, Roskilde, Denmark) were coated with ASF (0.1 µM, 50 µL per well) in PBS buffer (50 mM NaH_2_PO_4_, 150 mM NaCl, pH 7.5) overnight. Then the wells were blocked with BSA (2% w/v) diluted in PBS (1 h, r.t.). Afterwards, a mixture of the respective glyconanomaterial **24**–**39** in varying concentrations together with Gal-3 (total volume 50 µL; 6.5 µM final Gal-3 concentration) in EPBS buffer (50 mM NaH_2_PO_4_, 150 mM NaCl, 2 mM ethylenediaminetetraacetic acid, pH 7.5) were added and incubated for 2 h. Extensive rinsing of wells after every step with PBS containing Tween 20 (0.05% v/v) was performed. Bound Gal-3 was detected using anti-His_6_-IgG1 antibody from mouse conjugated with horseradish peroxidase (Roche Diagnostics, Mannheim, Germany) diluted in PBS (1/1000, 50 µL per well, 1 h, r.t.). TMB One substrate solution (Kem-En-Tec, Taastrup, Denmark) was utilized to react with IgG-conjugated peroxidase. The reaction was stopped by adding 3 M HCl (50 µL). The signal of bound Gal-3 was quantified colorimetrically (450 nm) using Sunrise absorbance microplate reader (Tecan Group Ltd, Switzerland). The acquired data were analyzed by non-linear regression (dose response-inhibition-variable slope) using GraphPad Prism 7.01 (GraphPad software, Inc.).

#### Surface plasmon resonance (SPR)

SPR measurements were performed at 25 °C using a ProteOn XPR36 Protein Interaction Array System (Bio-Rad) as described previously [16]. Briefly, the purified Gal-3-AVI protein (5 µg/mL) was captured on a neutravidin-coated NLC sensor chip (Bio-Rad) at a flow rate of 30 µL/min in a running buffer of PBS supplemented with 0.005% Tween-20 (PBS-T). Then, the serially-diluted glycopolymers (10–0.625 µM) in PBS-T were injected over the immobilized Gal-3-AVI at a flow rate of 30 µL/min. The Gal-3-AVI surface was regenerated by the injection of 50 mM HCl for 60 s at a flow rate of 30 µL/min. The resulting sensograms (binding curves) were obtained by subtracting the measured data from non-specific binding and baseline drift by interspot referencing and blank injection, respectively.

## Results and discussion

Recently, we have published a pioneer study showing the potential of chitooligosaccharide-containing HPMA-copolymers to obtain a nanomaterial with high affinity to wheat germ agglutinin [39]. In the present study we applied the acquired knowledge in the design and synthesis of therapeutically applicable oligosaccharide-HPMA copolymer nanomaterials suitable for selective inhibition of Gal-3 and possibly also for advanced actively-targeted controlled drug delivery to Gal-3-overexpressing tissue. We have evaluated in detail the structure-binding activity relation to the therapeutically relevant lectin, Gal-3, to produce a highly selective glyconanomaterial with good Gal-3 targeting ability.

### Chemoenzymatic synthesis of functionalized glycans

**Scheme 1:**
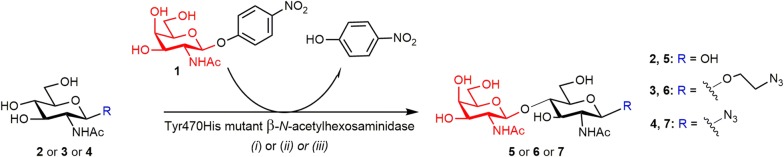
Enzymatic syntheses of LacdiNAc standard (**5**) and of functionalized disaccharides **6** and **7**. LacdiNAc (**5**) was prepared from donor **1** and GlcNAc (**2**) under the catalysis by Tyr470His mutant of the β-*N*-acetylhexosaminidase from *Talaromyces flavus* in (*i*), McIlvaine buffer pH 5.0/acetonitrile, 9/1, v/v, 45 °C, 4 h; disaccharide **6** was prepared from donor **1** and acceptor **3** under the catalysis by Tyr470His mutant of the β-*N*-acetylhexosaminidase from *Talaromyces flavus* in (*ii*), McIlvaine buffer pH 5.0/acetonitrile, 9/1, v/v, 45 °C, 5 h; disaccharide **7** was prepared from donor **1** and acceptor **4** using the same enzyme in (*iii*), McIlvaine buffer pH 5.0/acetonitrile, 9/1, v/v, 45 °C, 6.5 h

For a high-yielding coupling of the LacdiNAc epitope to HPMA copolymers by CuAAC, we employed two different functionalities: 2-azidoethyl group as in 2-azidoethyl 2-acetamido-2-deoxy-β-d-glucopyranoside (**3**) and in the respective disaccharide **6**, and a more rigid azido-moiety directly attached to the C-1 of GlcNAc as in 2-acetamido-2-deoxy-β-d-glucopyranosyl azide (**4**) and in the respective disaccharide **7**. The functionalized glycosyl acceptors **3** and **4** were prepared by standard chemical procedures [38, 39]. For the regioselective attachment of the GalNAc moiety under the formation of the LacdiNAc epitope, we employed transglycosylation reactions catalyzed by Tyr470His mutant of the β-*N*-acetylhexosaminidase from *Talaromyces flavus*. β-*N*-Acetylhexosaminidases are typical of intrinsic dual activity (processing both β-GlcNAc and β-GalNAc moieties) [42] and they have already proved their synthetic potential in the preparation of numerous functionalized oligosaccharides [43–45]. Here we employed our mutant transglycosidases constructed from the *T. flavus* β-*N*-acetylhexosaminidase by site-directed mutagenesis [34]. Due to a greatly suppressed hydrolytic activity, these mutants catalyze the oligosaccharide product formation in a much higher yield than the wild-type β-*N*-acetylhexosaminidases. The synthetic potential of these transglycosidases was previously exploited only in the transfer of the GlcNAc moiety [39]. Here, for the first time, their synthetic utility was also demonstrated in the synthesis GalNAc-terminated glycans. Two of these transglycosidases were tested for this aim: Tyr470Phe and Tyr470His mutants. The Tyr470Phe mutant was found to also process the azidoethyl (**3**) and the azido (**4**) acceptors as substrates—to such an extent that the final product conversion and reaction selectivity were deemed unsatisfactory. Therefore, we opted for the Tyr470His mutant for the preparation of **6** and **7** (Scheme 1). Since *p*NP-GalNAc donor (**1**) has quite a low solubility in water (saturated concentration ca. 10 mM at 60 °C), various concentrations of acetonitrile co-solvent as well as different temperatures were tested in order to maximize the reaction yield. The synthesis of disaccharide **7** was complicated by the formation of unwanted by-product 2-acetamido-2-deoxy-β-d-glucopyranosyl-(1 → 4)-2-acetamido-2-deoxy-β-d-glucopyranosyl azide (for the structural information see Additional file 1: Figure S4). This by-product was formed as a result of partial cleavage and transfer of azido acceptor **4**, which could not be completely abolished under any conditions tested. Glycosidases are known to cleave and transfer glycosyl using glycosyl azide as a donor [38, 46], though at a much lower rate than with aryl glycosides. Due to its high structural similarity, this by-product was separable by gel chromatography only partially, which resulted in a lower preparative yield of **7** compared to **6**. Despite this, the isolated yields of both disaccharides **6** and **7** were good (35-48%). The LacdiNAc standard **5** was prepared analogously to **6**. The purity of compounds **5**-**7** was verified by HPLC (see Additional file 1: Figs S8–S10) and their structure was confirmed by HRMS and ^1^H and ^13^C NMR (see Additional file 1: Tables S1–S3 and Figs. S1–S3 and S5–S7).

### Synthesis of polymer precursors and glyconanomaterials

**Scheme 2:**
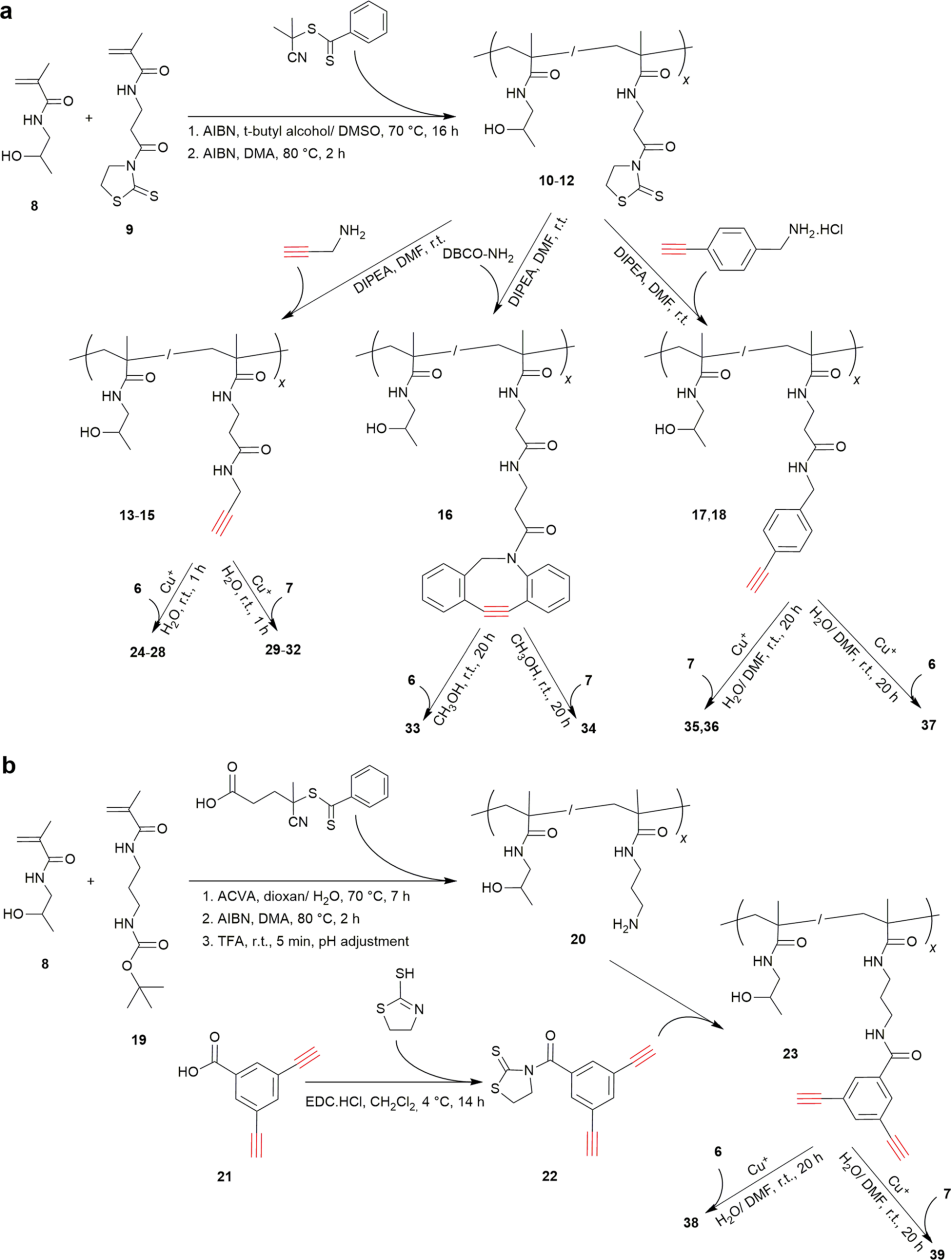
**a** Synthesis of copolymers **13**–**18** and glyconanomaterials **24**–**37**; **b** Synthesis of copolymer **23** and glyconanomaterials **38** and **39**

In the scope of this structure–activity relationship study, we prepared a range of functionalized copolymers bearing alkyne groups, which varied in their neighborhood and content in the polymer. Besides copolymers bearing propargyl groups (**13**–**15**), described previously, we also synthesized copolymers with one (**17**, **18**) or two ethynyl groups (**23**) bound to an aromatic ring or a copolymer with a triple bond bound to a complex of neighbouring aromatic rings (**16**). The content of triple bonds available for CuAAC of oligosaccharides ranged from 5 to 30 mol. %. The respective copolymer precursors **10**–**12** were prepared by controlled radical RAFT polymerization and contained ca. 11, 21 or 31 mol.  % of TT groups, respectively. The reaction conditions were optimized to obtain comparable molecular weights (*M*_n_ ca. 25,000 g/mol) and a narrow dispersity (*Ð* ca. 1.1). The triple bonds were introduced using the aminolytic reaction of the respective bifunctional azide-containing reagent with TT groups of the polymer precursors (**10**–**12**) to afford functionalized copolymers **13**–**18** and **23** (Scheme 2). The characteristics of all copolymers such as molar mass and the content of each spacer are shown in Table 1. Notably, the introduction of alkyne-containing moieties in the polymer precursors led to a significant decrease in its hydrophilicity. As a result, the copolymers with a high content of propargyl (29 mol. %; **15**), DBCO (8 mol. %, data not shown) or ethynylphenyl (20 mol. %; **18**) groups were not soluble in water but in methanol.

**Table 1:** Characteristics of polymer precursors used for the conjugation of LacdiNAc epitope

Compound	Prepared from	Precursor for glycopolymer	Functional groups		*M*_n_ (g/mol)^a^	*Ð* ^a^
			Type	Content (mol. %)		
**10**	**8**, **9**	–	TT	11.4	22,900	1.06
**11**	**8**, **9**	–	TT	20.5	25,300	1.13
**12**	**8**, **9**	–	TT	31.0	27,700	1.04
**13**	**10**	**24**, **25**, **29**, **30**	Propargyl	10.3	21,400	1.07
**14**	**11**	**26**, **31**	Propargyl	20.1	22,700	1.14
**15**	**12**	**27**, **28**, **32**	Propargyl	29.4	20,700	1.18
**16**	**10**	**33**, **34**	DBCO	5.4	21,500	1.22
**17**	**10**	**35**	Ethynylphenyl	9.9	21,200	1.07
**18**	**11**	**36**, **37**	Ethynylphenyl	20.2	27,400	1.11
**20**	**8**, **19**	–	NH_2_	8.5	27,500	1.08
**23**	**20**	**38**, **39**	Diethynylphenyl	6.8^b^	31,700	1.11

^a^Molecular weight (*M*_n_) and dispersity (*Ð*) of polymer precursors were determined using GPC with RI and MALS detection using TSKgel Super SW3000 column and methanol/0.3 M sodium acetate buffer, pH 6.5 (4/1, v/v) as the mobile phase

^b^6.8 mol. % is the content of linker molecule corresponding to 13.6 mol. % of ethynylphenyl moieties available for CuAAC

The functionalized copolymers were then conjugated with azido-functionalized disaccharides **6** and **7**. The varying content of alkyne groups enabled to attach a largely varying amount of LacdiNAc glycan in order to analyze the impact of the glycan density in the glycopolymer on the binding efficacy to Gal-3. Admittedly, the highest concentrations of glycans conjugated to the polymer used here would probably be difficult for in vivo application since the glyconanomaterial pharmacokinetics may be influenced. However, in the present in vitro structure-affinity relationship study, we opted for a larger choice of studied glycan densities in order to reach a deeper understanding of the system.

**Scheme 3:**
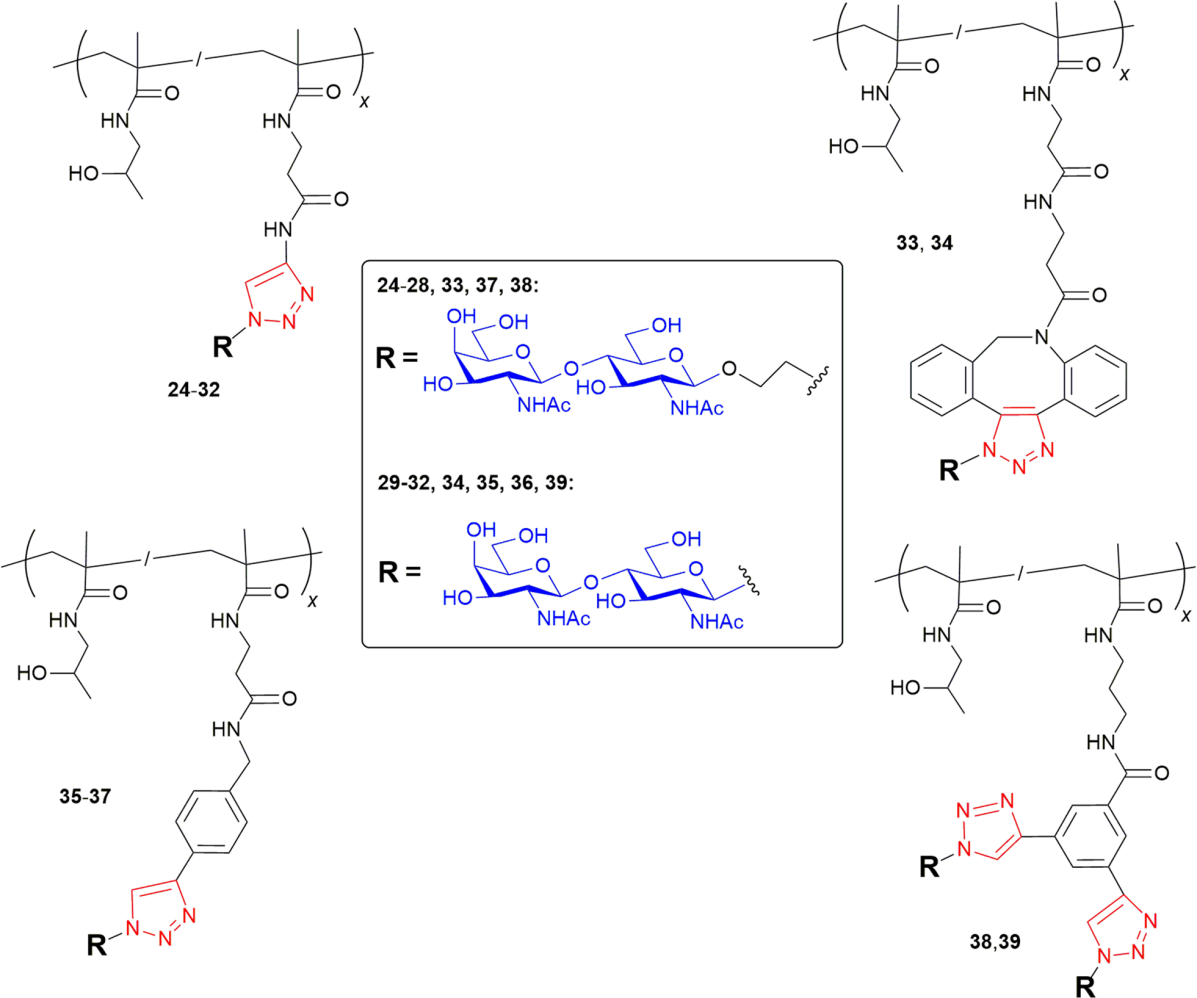
Structures of glyconanomaterials **24**–**39**. The contents of LacdiNAc disaccharide in the glyconanomaterials are given in Table 2

In total, sixteen glyconanomaterials of HPMA copolymers with functionalized saccharides **6** or **7** were prepared (**24**–**39**), which contained 3-29 mol. % of LacdiNAc epitope conjugated through various linkers (propargyl, DBCO, ethynylphenyl and bivalent diethynylphenyl). Fourteen of them were synthesized by Cu(I)-mediated click reaction and two by means of a copper-free click reaction (**33** and **34**). The respective structures are depicted in Scheme 3. The use of click chemistry for attaching intact functionalized oligosaccharides to a multivalent carrier is generally much more advantageous, better defined and higher yielding than attempts to enzymatically glycosylate a multivalent precursor, which often afford a partially glycosylated by-product [47]. The molecular weights of the prepared glyconanomaterials increased when compared to the respective polymer precursors as a consequence of the presence of the saccharide moieties attached to the polymer structure (cf. respective data in Tables 1, 2). As expected, the attachment of saccharides did not influence polymer dispersity. The conjugation of disaccharides to polymer precursors via azide-alkyne cycloaddition reaction was nearly quantitative as demonstrated in the formation of the triazole moiety signal in NMR spectra. The course of click reaction of azido function of **6** or **7** with propargyl groups of copolymers **13**–**15** was observed in situ by NMR spectroscopy. An analogous experiment was also performed for copolymers with alkyne-phenyl moieties (**17**, **18**, **23**). While the propargyl groups were clicked within minutes yielding glyconanomaterials **24**–**32**, the click reaction to alkyne in the vicinity of the aromatic ring was much slower—a near-to-quantitative conversion was observed after 3 h at r.t. There were no differences between the reactivity of the azido function group attached to the glycan directly (as in **6**) or via a short ethyl spacer (as in **7**). In the case of copolymer **23** bearing bivalent diethynylphenyl spacers, we used a 1.25-molar excess of saccharide to the polymer alkyne groups in order to modify all triple bonds available. Although copolymers with a higher molar content of alkynyl groups (**15**, **18)** and the copolymer with 8 mol. % DBCO (data not shown) were not water-soluble as mentioned above, the respective conjugates with hydrophilic LacdiNAc glycans were well soluble in water. The only exception was the glyconanomaterial prepared from the copolymer carrying 8 mol. % DBCO (data not shown). In this case, neither the copolymer nor the respective glycomaterial were water soluble and therefore they were excluded from the study. All other glyconanomaterials (**24**–**39**) were eligible for further in vitro testing of the binding activity to Gal-3.

**Table 2:** Competitive inhibition of Gal-3 binding to ASF by glycopolymers **24**–**39** determined by ELISA

Compound	*M*_n_ (g/mol)^a^	*Ð* ^a^	Sugar motif	*n* ^b^	IC_50_/glycan (μM)	IC_50_ (μM)	*r*_p_^c^	*r*_p_/*n*^d^
**Lactose**	342.3	n.a.	Galβ1, 4Glc	1	n.a.	137 ± 26	1	1
**LacNAc**	383.4	n.a.	Galβ1, 4GlcNAc	1	n.a.	53 ± 16	1	1
**LacdiNAc** ^e^	424.4	n.a.	GalNAcβ1, 4GlcNAc	1	n.a.	64 ± 20	1	1
**24**	25,200	1.08	LacdiNAc-*O*Et-triazole	6.6 (4.4%)	62.9 ± 9.6	9.5 ± 1.5	7	1.0
**25**	27,300	1.10	LacdiNAc-*O*Et-triazole	11.9 (8.2%)	66.5 ± 14.9	5.6 ± 1.2	11	1.0
**26**	34,270	1.18	LacdiNAc-*O*Et-triazole	20.3 (12.5%)	66 ± 17	3.25 ± 0.85	20	1.0
**27**	34,000	1.20	LacdiNAc-*O*Et-triazole	23.9 (16.3%)	94 ± 19	3.92 ± 0.81	16	0.7
**28**	38,600	1.15	LacdiNAc-*O*Et-triazole	37.2 (29.0%)	126 ± 22	3.39 ± 0.60	19	0.5
**29**	21,700	1.10	LacdiNAc-*N*-triazole	3.7 (2.7%)	32.7 ± 7.9	8.6 ± 2.1	8	2.0
**30**	24,100	1.10	LacdiNAc-*N*-triazole	10.6 (8.1%)	27.5 ± 4.6	2.59 ± 0.44	25	2.3
**31**	30,800	1.12	LacdiNAc-*N*-triazole	18.2 (12.0%)	62 ± 20	3.4 ± 1.1	19	1.0
**32**	38,900	1.39	LacdiNAc-*N*-triazole	36.1 (24.8%)	128 ± 17	3.53 ± 0.46	18	0.5
**33**	31,500	1.07	LacdiNAc-*O*Et-DBCO	9.6 (5.7%)	54.2 ± 5.9	5.65 ± 0.69	11	1.2
**34**	28,600	1.06	LacdiNAc-*N*-DBCO	5.2 (3.0%)	84 ± 19	16.2 ± 3.7	4	0.8
**35**	28,300	1.18	LacdiNAc-*N*-triazole-Ph	5.6 (3.2%)	31.5 ± 0.85	5.62 ± 0.15	11	2.0
**36**	51,700	1.18	LacdiNAc-*N*-triazole-Ph	34.7 (15.7%)	139 ± 54	4.0 ± 1.5	16	0.5
**37**	43,400	1.24	LacdiNAc-*O*Et-triazole-Ph	29.9 (17.2%)	101 ± 20	3.37 ± 0.66	19	0.6
**38**	48,600	1.30	(LacdiNAc-*O*Et-triazole)_2_Ph	28.0 (12.3%)	98 ± 20	3.5 ± 0.72	18	0.6
**39**	46,100	1.10	(LacdiNAc-*N*-triazole)_2_Ph	29.8 (14.0%)	76.8 ± 16.9	2.58 ± 0.57	25	0.8

n.a. not applicable, Et, ethyl; Ph, phenyl

^a^Molecular weight (*M*_n_) and dispersity (*Ð*) of polymers were determined using GPC with MALS and RI detection. Glycopolymers **28** and **32** were analyzed using Superose 12 column and 0.3 M sodium acetate buffer, pH 6.5 as the mobile phase; otherwise TSKgel Super SW3000 column and methanol/0.3 M sodium acetate buffer, pH 6.5 (4/1, v/v) as the mobile phase was employed. For monovalent standards, we give *M*_w_ (g/mol)

^b^Average number of glycans per polymer chain (glycan content, mol. %); *n* = 1, monovalent standard

^c^Relative potency, i.e. IC_50_ (monovalent standard)/IC_50_ (multivalent glycopolymer)

^d^Relative potency per one glycan

^e^Synthesis of LacdiNAc standard (Scheme 1) is described in the Experimental section

### Inhibitory potency of glyconanomaterials in ELISA assay

**Fig. 1:**
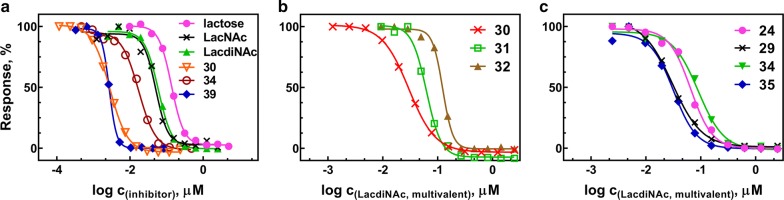
Competitive inhibition of Gal-3 binding to ASF by multivalent glyconanomaterials **24**–**39** as determined by ELISA. The following sample dose response inhibition curves are shown. **a** Inhibition by monovalent disaccharides lactose, LacNAc and LacdiNAc, by glyconanomaterials **30**, **39** (two best inhibitors in the series), and **34** (the worst inhibitor in the series). The sigmoidal curves yield the values of IC_50_ for the respective compounds. **b** Inhibition by glyconanomaterials **30**, **31**, and **32** (structural motif LacdiNAc-*N*-triazole). Here, the concentrations are calculated for the active substance of the glycomaterial—LacdiNAc glycan—and thus the curves yield the values of IC_50_ per glycan. The higher the density of glycans on the polymer backbone, the lower the inhibitory potency per glycan. **c** Inhibition by glyconanomaterials **24**, **29**, **34** and **35** (containing various linkers with a comparable molar content of LacdiNAc). Here again, the curves yield the values of IC_50_ per glycan. The type of attachment to the polymer backbone predestined the inhibitory potency per glycan

Glyconanomaterials **24**–**39** were tested for their ability to inhibit binding of Gal-3 to immobilized asialofetuin (ASF). ASF is a multivalent glycoprotein carrying three LacNAc-capped triantennary *N*-glycans and it is commonly used as a standard ligand in binding assays with human Gal-3. For the aims of ELISA assay, we utilized a soluble His-tagged construct of human Gal-3, which was recombinantly expressed in *E. coli* and purified by immobilized metal-ion affinity chromatography as described before [28, 36]. Gal-3 was incubated with increasing concentrations of the glyconanomaterials as competing ligands. The inhibition of Gal-3 binding to immobilized ASF conferred by respective glycomaterials was colorimetrically quantified using anti-His antibody conjugated to horseradish peroxidase and 3,3′,5,5′-tetramethylbenzidine (TMB) substrate. Lactose, LacNAc and LacdiNAc were used as positive controls. HPMA copolymer precursor **10** containing no carbohydrate showed no inhibition. The obtained dose–response sigmoidal inhibition curves (Fig. 1) were analyzed by non-linear regression and the respective inhibition constants (half-maximal inhibitor concentration IC_50_, i.e., the inhibitor concentration at which 50% inhibition of the binding of Gal-3 to immobilized ASF was reached) were calculated. They are listed in Table 2.

The data given in Table 2 show the influence of several structural parameters of studied glyconanomaterials on their inhibitory potential towards Gal-3: namely, the glycan density on the HPMA copolymer backbone (3–29 mol. %), the type of linker or glycan attachment (*O*-ethylazido- or azido-; propargyl or ethynylphenyl as well as the mode of glycan presentation (monovalent vs. bivalent linker).

The comparison of glyconanomaterials with the same structural motif and varying glycan densities (cf. compound series **24**–**28** and **29**–**32**) revealed that the optimum glycan concentration is in the range of 8–12 mol. % (compounds **26** and **30**). Thus, we could reach the highest relative inhibitory potency within the same structural motif (**26** and **30** are 20 and 25-times stronger inhibitors, respectively, that the monovalent LacdiNAc). When the glycan content on the polymer backbone was further increased (therewith shortening the mutual distance of glycans), the inhibitory potency stagnates or even slightly decreases; cf. IC_50_ per glycan within the series **24**–**28** and **29**–**32** (increasing IC_50_ reflects weaker inhibition). This trend is more pronounced in the series **29**–**32** where LacdiNAc is directly attached by the azido-moiety (cf. conjugates **30** and **32**). Clearly, this relatively rigid type of attachment of the glycan to the polymer backbone is more sterically hindered than the *O*-linked 2-ethylazido moiety (conjugates **24**-**28**). On the other hand, the presence of the rigid triazole directly linked to the glycan (i.e., LacdiNAc-*N*-triazole) apparently brings additional improvement in inhibitory potency: **30** is more than two-times stronger inhibitor than **25**, which has a comparable glycan content and differs only in the attachment of LacdiNAc through azido- or *O*-ethylazido functionality, respectively. This may be explained by structural relations in the binding pocket of Gal-3 carbohydrate recognition domain as revealed by molecular modelling and crystallization [25]. The aglycon bound to the C-1 at the glycan reducing end accommodates into the so-called “subsite E” where it may interact with Arg186. The presence of an arene cycle rich in π-electron density in this position thus brings additional affinity enhancement to the Gal-3-glycan interaction. However, the structure and type of this aglycon is quite a sensitive parameter since the use of a phenyl linker (leading to LacdiNAc-*N*-triazole-phenyl motif in **35**) did not bring further improvement in binding compared to **29** (comparable glycan content with LacdiNAc-*N*-triazole motif). Moreover, an even larger DBCO linker (resulting in LacdiNAc-*N*-DBCO in **34**) caused a decrease in binding potency of the resulting glycomaterial (**34** was a more than 6-times worse inhibitor than **30**). With the DBCO linker, the *O*-ethylazido functionality was a more successful combination (cf. LacdiNAc-*O*Et-DBCO in **33**). However, the DBCO linker was very badly water soluble, and therefore incompatible with the water-soluble HPMA copolymers.

The use of the bivalent linker, leading to (LacdiNAc-*O*Et-triazole)_2_-phenyl in **38** and (LacdiNAc-*N*-triazole)_2_-phenyl in **39**, addresses the question if a pairwise presentation of glycans conjugated through a bivalent linker can increase the conjugate inhibitory potency. In the case of *O*-ethyl-bound LacdiNAc, no apparent positive effect was shown in ELISA as **38** with bivalent linker has the same IC_50_ as **37** with the monovalent phenyl linker. The pairwise glycan distribution on the bivalent linker seems to be more successful in the case of *N*-linked **39**, which exhibited an enhanced inhibitory potency compared to the counterpart **36** with the monovalent linker. In sum, the best ligand in the series considering the relative potency per glycan is the glyconanomaterial **30** decorated with LacdiNAc-*N*-triazole moieties. With an IC_50_ of 2.5 μM it shows the highest relative potency per glycan in the series—*r*_p_/*n* = 2.3 compared to the monovalent LacdiNAc standard, which means that one LacdiNAc presented on the polymer in this structural arrangement is 2.3-times more potent than the respective monovalent standard. This is a good result for a disaccharide epitope and especially good for the LacdiNAc epitope, which displayed hardly any multivalence effect on a BSA scaffold [16]. Therefore, the triazole-*N*-glycan statistically distributed along the HPMA copolymer in ca. 8 mol. % content is a good starting point for the synthesis of glycan-decorated HPMA-copolymer-based nanomaterials of the second generation. Another outstanding conjugate is compound **39** featuring a pairwise distribution of *N*-linked LacdiNAc moieties through a bivalent phenyl linker. Though it is less efficient concerning the relative potency per glycan, this conjugate exhibited an IC_50_ of 2.6 μM, comparable to the best conjugate **30**.

### Binding kinetics of selected glyconanomaterials to Gal-3

**Fig. 2:**
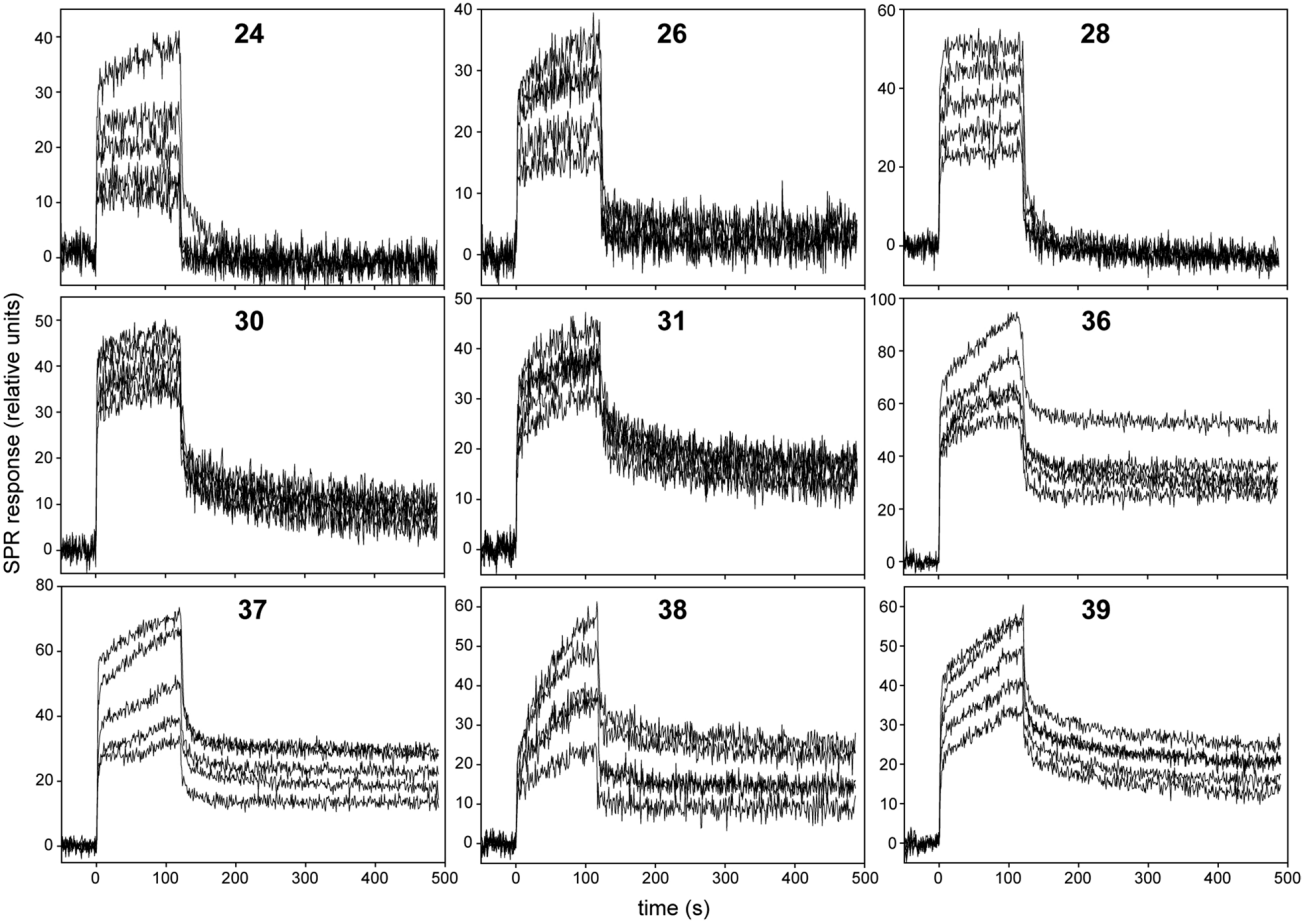
SPR analysis of kinetics of the interactions between the immobilized Gal-3-AVI and glyconanomaterials **24**, **26**, **28** (carrying the LacdiNAc-*O*Et-triazole motif), **30**, **31** (the LacdiNAc-*N*-triazole motif), **36**, **37** (the LacdiNAc-triazole-phenyl motif) **38**, **39** (the (LacdiNAc-triazole)_2_-phenyl motif). Each of the sensograms represents “one-shot kinetics” data obtained with two-fold dilutions (10**–**0.625 µM) of the respective glyconanomaterial

The kinetics of interaction of Gal-3 with nine selected glycomaterials (**24**, **26**, **28**, **30**, **31**, **36**–**39**) were monitored by SPR. To perform the SPR measurements, we employed our recently published SPR approach, in which we assessed the interaction of poly-*N*-acetyllactosamine (LacNAc) neo-glycoproteins to an immobilized Gal-3 containing the biotinylated AviTag sequence (Gal-3-AVI) [16]. The Gal-3-AVI construct is a fusion protein of Gal-3 with the AviTag peptide sequence (GLNDIFEAQKIEWHE) that serves as a target for biotin ligase (BirA) and enables enzymatic conjugation of a single biotin molecule to the lysine residue of the AviTag sequence. The purified, in vivo biotinylated Gal-3-AVI protein was captured to a neutravidin-coated sensor chip at a coupling level of 200 relative units (RU) and the binding of glycomaterials to Gal-3-AVI was probed by repeated injections of serially-diluted samples of glycomaterials (0.625–10 µM concentrations) at a flow rate of 30 µl/min. Typical results of real time interaction kinetics are presented in Fig. 2.

The binding curves show that the overall SPR response is relatively low, indicating that nanomolar concentrations of glycomaterials are not sufficient to saturate the Gal-3-AVI binding sites. Therefore, apparent dissociation constants for these interactions are likely to be in micromolar range. Regrettably, high concentrations of glyconanomaterials (> 10 µM) yielded very high non-specific binding that prevented the exact determination of the interaction kinetics at high concentrations of the analytes. Moreover, other tested strategies of Gal-3 immobilization, such as covalent immobilization through amine coupling chemistry or capturing of the His-tagged Gal-3 to a Ni^2+^-nitrilotriacetate (Ni–NTA) surface, did not yield any useful SPR data, which is in good agreement with our previous results [16]. Regardless of the low quality SPR data, certain information about the interaction kinetics of individual glycomaterials could be deduced from the binding curves. As shown in Fig. 2, the interaction of Gal-3-AVI with glycomaterials **24**, **26**, and **28**, carrying the LacdiNAc-Et*O*-triazole motif, was characterized by a rapid on/off kinetics (association and dissociation, respectively) that resembles the interaction kinetics of binding of LacdiNAc or other monovalent disaccharides to Gal-3. In contrast, two pairs of glycopolymers **30** and **31** as well as **36** and **37**, carrying the LacdiNAc-*N*-triazole or LacdiNAc-triazole-phenyl motif, respectively, gave rapid on-rate kinetics accompanied with significantly slower off-rates. On top of that, both the on- and off-rate kinetics of the glycomaterials **38** and **39**, carrying bivalent phenyl linkers with two LacdiNAc-triazole moieties, were very slow, giving rise to a biphasic-like binding curve of the SPR sensograms. These results imply, in correlation with ELISA assays, that the presence of an aromatic moiety (e.g., triazole) directly attached to the glycan chain reinforces the binding of the glyconanomaterial to Gal-3 (compounds **28**, **30**), and that elongation of the aromatic aglycon by a phenyl residue (compounds **36**, **37**) does not further improve but maintains the binding properties of the glycan. Furthermore, the kinetic analysis by SPR indicates that a pairwise glycan distribution in compounds **38** and **39** (i.e., pairs of LacdiNAc glycans are attached to the polymer backbone through the bivalent phenyl linker) confers the most favorable binding to Gal-3.

### Glyconanomaterial physico-chemical properties

The solution properties and interaction with blood plasma content of materials destined for the in vivo application are crucial parameters determining the applicability of the novel polymer system. Therefore, we measured the hydrodynamic radii (*R*_H_) of selected glyconanomaterials (**24**–**39**) by dynamic light scattering to determine if the carbohydrate portion had any impact on their behavior in solution (see Additional file 1: Table S4). The hydrodynamic radius *R*_H_, reflecting the size and shape of the polymer chain in the medium, was ca. 4 nm in all glyconanomaterials tested and was not influenced by the presence of conjugated saccharide when comparing glycomaterials **29**–**32**, carrying the same LacdiNAc-*N*-triazole motif in varying concentration, with their copolymer precursor **13**. The size of glycomaterials **38** and **39** containing a rather hydrophobic bivalent diethynylphenyl spacer (with the (LacdiNAc-triazole)_2_-phenyl motif) was also comparable (*R*_H_ of ca. 4.5 nm). Thus, we can conclude that the tested copolymers formed polymer coils similar to their precursor counterparts and that the glycans did not change the behavior of the glyconanomaterial in solution. Since it is known that amphiphilic copolymers may possibly bind plasma proteins via hydrophobic interaction, we incubated copolymers **13**, **29**-**32**, **38**, and **39** with BSA (50 mg/mL) to simulate the plasma protein environment, for 1 h. We did not observe any formation of aggregates and the size of BSA remained unchanged. Therefore, we conclude that the presence of the carbohydrate portion in our glycomaterials does not dramatically alter their physico-chemical behavior in the medium and do not enhance any non-specific interaction with the most common protein of the blood plasma. For the aim of future in vivo applications, further experiments on this issue may be needed, which are, however, beyond the scope of this study.

## Conclusions

In this work we have described novel polymer glyconanomaterials tailored for specific Gal-3 targeting, based on the combination of selective LacdiNAc disaccharide motif and biocompatible HPMA copolymer. We evaluated in detail the impact of various structural parameters of the glyconanomaterials on their affinity to Gal-3. The LacdiNAc epitope was prepared in a high-yield one-step reaction using our mutant β-*N*-acetylhexosaminidases. Since the synthesis of complex defined oligosaccharide epitopes is very challenging, the LacdiNAc disaccharide represents a compromise between efficiency and selectivity on the one hand and synthetic simplicity and scalability on the other. By choosing the optimum glycan concentration and linker we were able to shift the inhibitory potency (affinity to Gal-3) by up to one order of magnitude. We have found strong influence of several structural parameters on the inhibitory potential of studied glycomaterials towards Gal-3. We can conclude that the 8–12 mol. % of glycan epitopes is the optimal amount leading to the multivalence effect and that the glycans are optimally presented via the *N*-triazole linker. The SPR data confirmed that the presence of an aromatic moiety (e.g., triazole) directly attached to the glycan chain reinforced the conjugate binding to Gal-3. Our study shows the potential of the prepared glyconanomaterials for targeting and/or inhibition of the therapeutically relevant Gal-3 lectin. It is a good starting point for the development of new efficient inhibitors of Gal-3 based on the biocompatible HPMA polymer scaffold and it implies the possibilities of a future therapeutic utility of these hybrid polymer glyconanomaterials.

## Additional file

**Additional file 1.** Structural characterization of disaccharides **5**–**7** (NMR data and spectra, MS spectra, HPLC chromatograms of preparations), procedures of the synthesis of polymer precursors and glyconanomaterials, structural characterization of selected compounds (NMR spectra), hydrodynamic radii of selected compounds.

## Abbreviations

ACVA: 4,4′-azobis(4-cyanopentanoic acid)
AIBN: 2,2′-azobisisobutyronitrile
APMA-Boc: *N*-(3-*tert*-butoxycarbonyl-aminopropyl)methacrylamide
ASF: asialofetuin
BSA: bovine serum albumin
CRD: carbohydrate recognition domain
CTA: 2-cyanopropan-2-yl dithiobenzoate
CuAAC: Cu(I)-catalyzed azide–alkyne cycloaddition
DBCO: dibenzylcyclooctyne
DIPEA: *N*,*N*-diisopropylethylamine
DMA: *N*,*N*-dimethylacetamide
DMF: *N*,*N*-dimethylformamide
DMSO: dimethyl sulfoxide
EDC.HCl: 1-ethyl-3-(3-dimethylaminopropyl)carbodiimide hydrochloride
ELISA: enzyme-linked immunosorbent assay
Gal-3: galectin-3
Gal-3-AVI: galectin-3 containing AviTag peptide sequence
HPLC: high-performance liquid chromatography
HPMA: *N*-(2-hydroxypropyl)methacrylamide
HRMS (ESI): electrospray ionization high resolution mass spectrometry
LacdiNAc: *N*,*N*′-diacetyllactosamine (GalNAcβ1,4GlcNAc)
LacNAc: *N*-acetyllactosamine (Galβ1,4GlcNAc)
MA-AP-TT: 3-(3-methacrylamidopropanoyl) thiazolidine-2-thione
NMR: nuclear magnetic resonance
*p*NP-GalNAc: *p*-nitrophenyl 2-acetamido-2-deoxy-β-d-galactopyranoside
*p*NP-GlcNAc: *p*-nitrophenyl 2-acetamido-2-deoxy-β-d-glucopyranoside
r.t.: room temperature
SPR: surface plasmon resonance
TFA: trifluoroacetic acid
