# Supplementary material for: Biocompatible glyconanomaterials based on HPMA-copolymer for specific targeting of galectin-3
Source: J Nanobiotechnology. 2018 Sep 20;16:73. doi: 10.1186/s12951-018-0399-1 (PMC6146777; doi:10.1186/s12951-018-0399-1)
Supplement: Supplementary file 1 — Additional file 1. Structural characterization of disaccharides 5–7 (NMR data and spectra, MS spectra, HPLC chromatograms of preparations), procedures of the synthesis of polymer precursors and glyconanomaterials, structural characterization of selected compounds (NMR spectra), hydrodynamic radii of selected compounds. [file 12951_2018_399_MOESM1_ESM.pdf]

# Supplementary Material

## Biocompatible glyconanomaterials based on HPMA-copolymer for specific targeting of galectin-3

P. Bojarová[1]*, M. R. Tavares[2], D. Laaf[3], L. Bumba[1], L. Petrásková[1], R. Konefał[2], M. Bláhová[2], H. Pelantová[1], L. Elling[3], T. Etrych[2], P. Chytil[2]* and V. Křen[1]

[1] Institute of Microbiology, Czech Academy of Sciences, Vídeňská 1083, Prague 4, CZ-14220, Czech Republic.
[2] Institute of Macromolecular Chemistry, Czech Academy of Sciences, Heyrovský Sq. 2, Prague 6, CZ -16206, Czech Republic.
[3] Laboratory for Biomaterials, Institute for Biotechnology and Helmholtz-Institute for Biomedical Engineering, RWTH Aachen University, Pauwelsstraße 20, Aachen, D-52074, Germany.

* Correspondence: bojarova@biomed.cas.cz; chytil@imc.cas.cz.
These authors contributed equally to this work.

### Abstract

**Background**: Galectin-3 (Gal-3) is a promising target in cancer therapy with a high therapeutic potential due to its abundant localization within the tumor tissue and its involvement in tumor development and proliferation. Potential clinical application of Gal-3-targeted inhibitors is often complicated by their insufficient selectivity or low biocompatibility. Nanomaterials based on *N*-(2-hydroxypropyl)methacrylamide (HPMA) copolymer are attractive for *in vivo* application due to their good water solubility and lack of toxicity and immunogenicity. Their conjugation with tailored carbohydrate ligands can yield specific glyconanomaterials applicable for targeting biomedicinally relevant lectins like Gal-3.

**Results**: In the present study we describe the synthesis and the structure-affinity relationship study of novel Gal-3-targeted glyconanomaterials, based on hydrophilic *N*-(2-hydroxypropyl)methacrylamide (HPMA) copolymers. HPMA copolymers decorated with varying amounts of Gal-3 specific epitope GalNAcβ1,4GlcNAc (LacdiNAc) were analyzed in a competitive ELISA-type assay and their binding kinetics was described by surface plasmon resonance. We showed the impact of various linker types and epitope distribution on the binding affinity to Gal-3. The synthesis of specific functionalized LacdiNAc epitopes was accomplished under the catalysis by mutant β-*N*-acetylhexosaminidases. The glycans were conjugated to statistic HPMA copolymer precursors through diverse linkers in a defined pattern and density using Cu(I)-catalyzed azide–alkyne cycloaddition. The resulting water-soluble and structurally flexible synthetic glyconanomaterials exhibited affinity to Gal-3 in low μM range.

**Conclusions**: The results of this study reveal the relation between the linker structure, glycan distribution and the affinity of the glycopolymer nanomaterial to Gal-3. They pave the way to specific biomedicinal glyconanomaterials that target Gal-3 as a therapeutic goal in cancerogenesis and other disorders.

**Keywords:** carbohydrate; galectin-3; glyconanomaterial; HPMA copolymer, surface plasmon resonance

## Table of Contents


# 1. LacdiNAc disaccharides – structural characterization

1.1. NMR data and spectra

1.2. MS spectra

1.3. HPLC chromatograms

# 2. Glyconanomaterials – synthesis and characterization

2.1. Synthesis of polymer precursors

2.2. Synthesis of glyconanomaterials

2.3. NMR spectra

2.4. Hydrodynamic radii

# 1. LacdiNAc disaccharides – structural characterization

## 1.1. NMR data and spectra

**Table S1** $^1H$ and $^{13}C$ NMR data of disaccharide **5** (700.13 MHz for $^1H$, 176.05 MHz for $^{13}C$, $D_2O$, 30 °C)

**Part A**, β-D-GalNAc-(1→4)-α-D-GlcNAc

| | Atom | $\delta_C$ | m. | $\delta_H$ | $n_H$ | m. | *J*[Hz] |
|---|---|---|---|---|---|---|---|
| **GlcNAc$^A$** | **1** | 90.69 | D | 4.976 | 1 | d | 3.3 |
| | **2** | 53.80 | D | 3.658 | 1 | dd | 10.7, 3.3 |
| | **3** | 69.59 | D | 3.687 | 1 | dd | 10.7, 7.8 |
| | **4** | 79.83 | D | 3.422 | 1 | dd | 10.0, 7.8 |
| | **5** | 70.22 | D | 3.67$^H$ | 1 | m | |
| | **6** | 60.32 | T | 3.572 | 1 | dd | 12.1, 2.3 |
| | | | | 3.454 | 1 | dd | 12.1, 4.8 |
| | **2-CO** | 174.70 | S | - | 0 | | |
| | **2-Ac** | 22.13 | Q | 1.820 | 3 | s | |
| **GalNAc$^B$** | **1** | 102.00 | D | 4.315 | 1 | d | 8.4 |
| | **2** | 52.84 | D | 3.719 | 1 | dd | 10.8, 8.4 |
| | **3** | 70.96 | D | 3.541 | 1 | dd | 10.8, 3.3 |
| | **4** | 67.87 | D | 3.728$^a$ | 1 | d | 3.3 |
| | **5** | 75.58 | D | 3.51$^H$ | 1 | m | |
| | **6** | 61.18 | T | 3.56$^H$ | 2 | m | |
| | **2-CO** | 175.00 | S | - | 0 | | |
| | **2-Ac** | 22.43 | Q | 1.850 | 3 | s | |

**Part B**, β-D-GalNAc-(1→4)-β-D-GlcNAc

| | Atom | $\delta_C$ | m. | $\delta_H$ | $n_H$ | m. | *J*[Hz] |
|---|---|---|---|---|---|---|---|
| **GlcNAc$^A$** | **1** | 95.10 | D | 4.485 | 1 | m | ΣJ = 8.3 |
| | **2** | 56.25 | D | 3.484 | 1 | m | |
| | **3** | 72.83 | D | 3.484 | 1 | m | |
| | **4** | 79.38 | D | 3.412 | 1 | m | |
| | **5** | 74.84 | D | 3.307 | 1 | ddd | 9.8, 5.5, 2.1 |
| | **6** | 60.43 | T | 3.614 | 1 | dd | 12.3, 2.1 |
| | | | | 3.432 | 1 | dd | 12.3, 5.5 |
| | **2-CO** | 174.97 | S | - | 0 | | |
| | **2-Ac** | 22.42 | Q | 1.818 | 3 | s | |
| **GalNAc$^B$** | **1** | 101.97 | D | 4.305 | 1 | d | 8.4 |
| | **2** | 52.80 | D | 3.710 | 1 | dd | 10.8, 8.4 |
| | **3** | 70.93 | D | 3.531 | 1 | dd | 10.8, 3.3 |
| | **4** | 67.87 | D | 3.727$^a$ | 1 | d | 3.3 |
| | **5** | 75.58 | D | 3.51$^H$ | 1 | m | |
| | **6** | 61.19 | T | 3.56$^H$ | 2 | m | |
| | **2-CO** | 175.01 | S | - | 0 | | |
| | **2-Ac** | 22.43 | Q | 1.848 | 3 | s | |

$^a$ ... might be interchanged; $^H$ ... HSQC readout

a

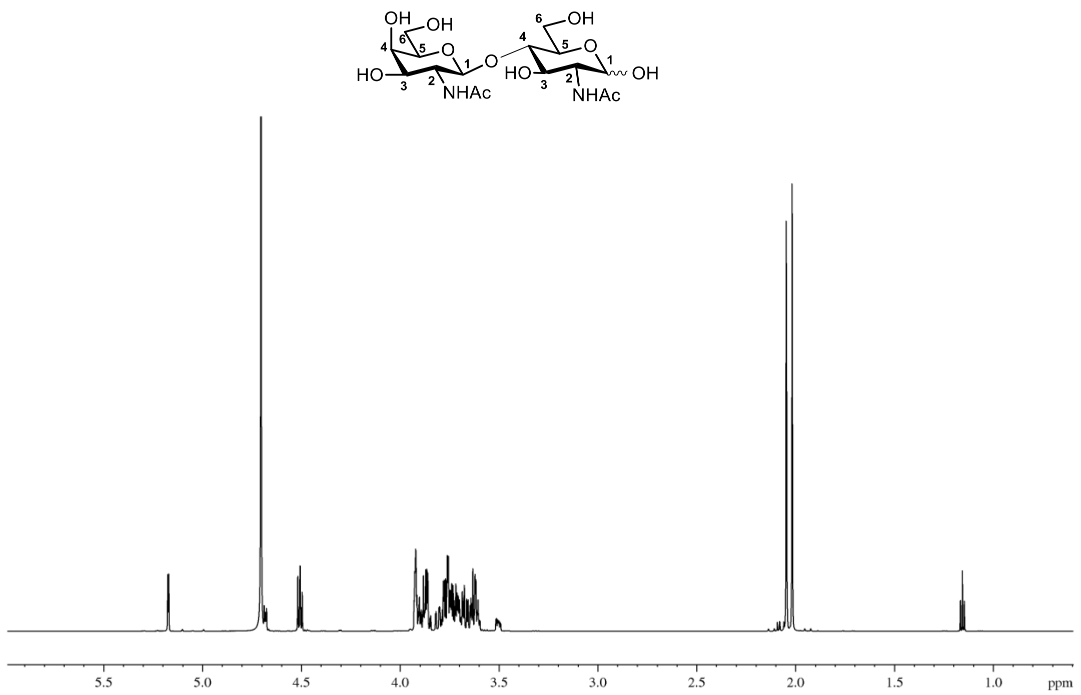

b

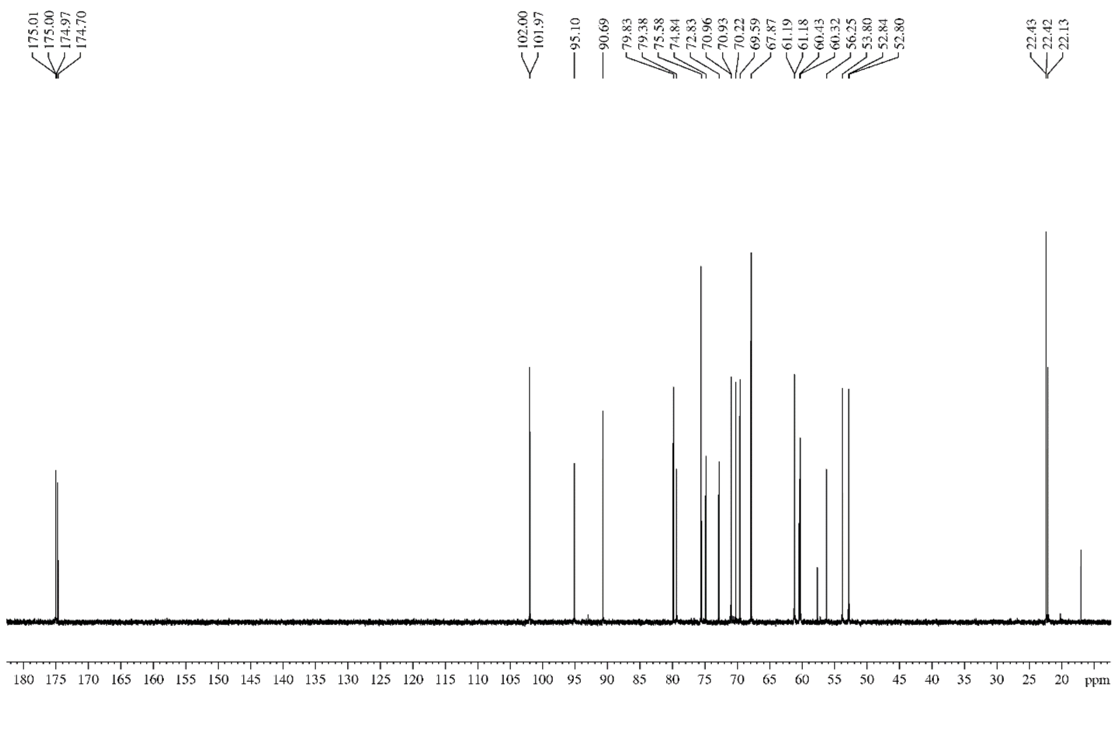

**Fig. S1** (a) $^{1}H$ and (b) $^{13}C$ NMR spectra of disaccharide **5** (700.13 MHz for $^{1}H$, 176.05 MHz for $^{13}C$, $D_2O$, 30 $^{o}C$).

**Table S2** $^{1}H$ and $^{13}C$ NMR data of disaccharide **6** (600.23 MHz for $^{1}H$, 150.94 MHz for $^{13}C$, $D_2O$, 25 °C).

| | **Atom** | $\delta_C$ | **m.** | $\delta_H$ | **n$_H$** | **m.** | ***J*[Hz]** |
|---|---|---|---|---|---|---|---|
| **spacer** | **1'** | 68.96 | T | 3.768 | 1 | ddd | 11.4, 5.6, 3.0 |
| | | | | 3.489 | 1 | ddd | 11.4, 7.6, 3.1 |
| | **2'** | 50.55 | T | 3.207 | 1 | ddd | 13.8, 7.6, 3.0 |
| | | | | 3.144 | 1 | ddd | 13.8, 5.6, 3.1 |
| **GlcNAc$^A$** | **1** | 101.16 | D | 4.307 | 1 | d | 8.3 |
| | **2** | 54.97 | D | 3.490 | 1 | dd | 10.4, 8.3 |
| | **3** | 72.78 | D | 3.439 | 1 | dd | 10.4, 8.3 |
| | **4** | 79.31 | D | 3.355 | 1 | dd | 9.8, 8.3 |
| | **5** | 74.75 | D | 3.254 | 1 | ddd | 9.8, 5.5, 2.2 |
| | **6** | 60.31 | T | 3.584 | 1 | dd | 12.2, 2.2 |
| | | | | 3.391 | 1 | dd | 12.2, 5.5 |
| | **2-CO** | 174.87 | S | - | 0 | | |
| | **2-Ac** | 22.44 | Q | 1.764 | 3 | s | |
| **GalNAc$^B$** | **1** | 101.92 | D | 4.248 | 1 | d | 8.5 |
| | **2** | 52.73 | D | 3.657 | 1 | dd | 10.9, 8.5 |
| | **3** | 70.86 | D | 3.475 | 1 | dd | 10.9, 3.3 |
| | **4** | 67.79 | D | 3.669 | 1 | dd | 3.3, 0.9 |
| | **5** | 75.52 | D | 3.450 | 1 | ddd | 8.2, 4.0, 0.9 |
| | **6** | 61.14 | T | 3.527 | 1 | dd | 11.8, 8.2 |
| | | | | 3.489 | 1 | dd | 11.8, 4.0 |
| | **2-CO** | 174.97 | S | - | 0 | | |
| | **2-Ac** | 22.37 | Q | 1.796 | 3 | s | |

a

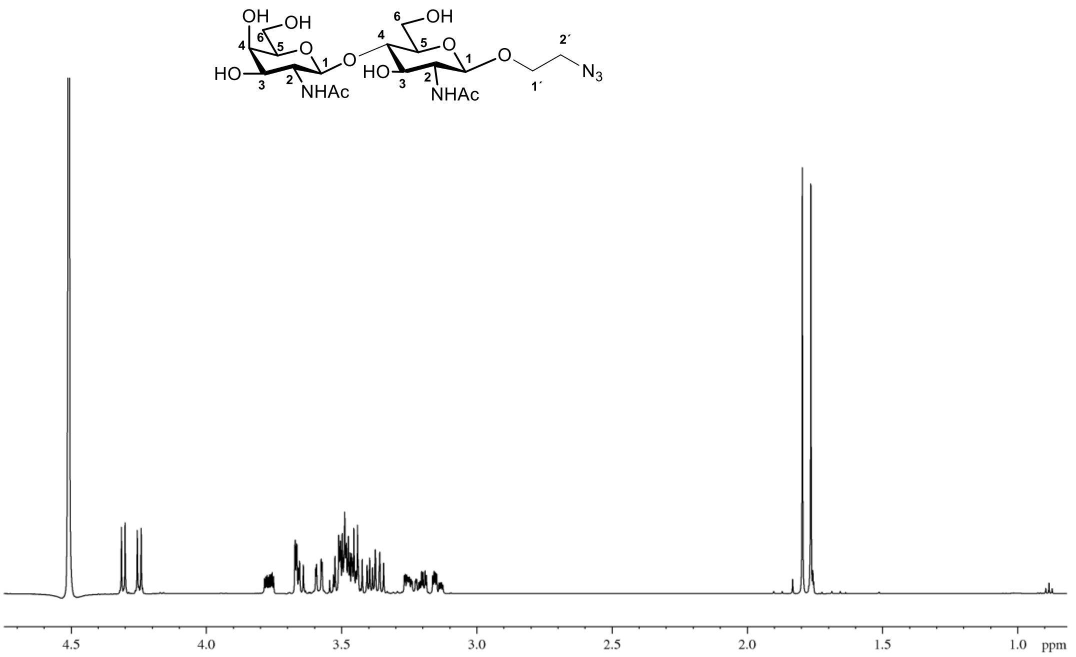

b

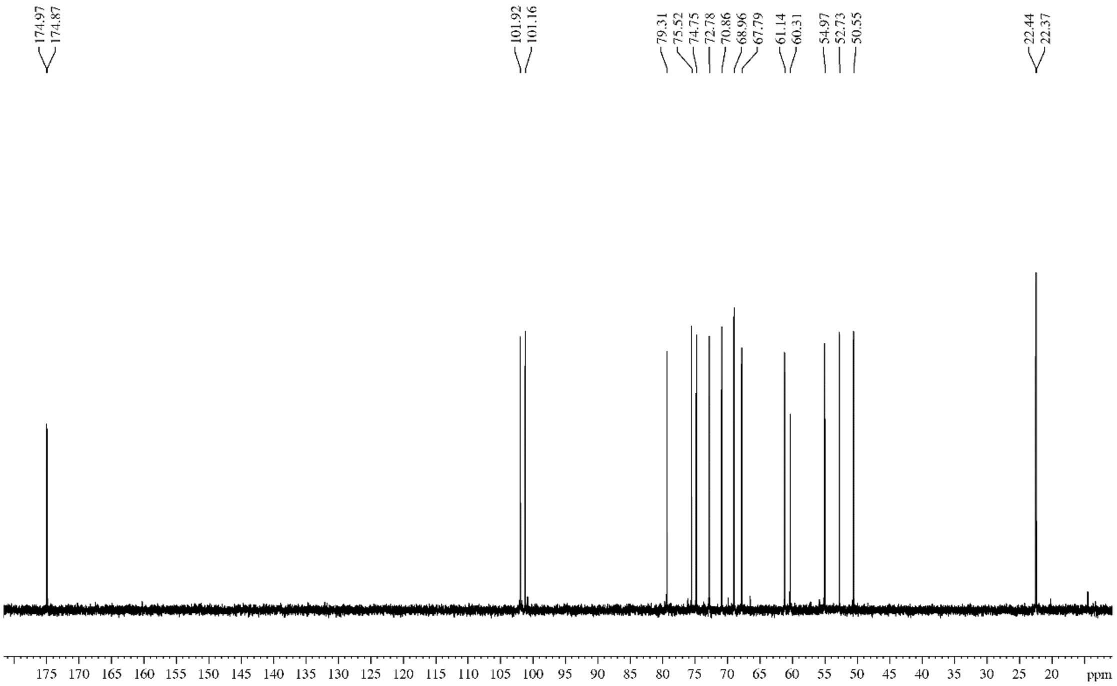


**Fig. S2** (a) $^{1}H$ and (b) $^{13}C$ NMR spectra of disaccharide **6** (600.23 MHz for $^{1}H$, 150.94 MHz for $^{13}C$, $D_2O$, 25 $^{o}C$).

**Table S3** $^{1}H$ and $^{13}C$ NMR data of disaccharide **7** (700.13 MHz for $^{1}H$, 176.05 MHz for $^{13}C$, $D_2O$, 30 $^{o}C$)

| | **Atom** | $\delta_C$ | **m.** | $\delta_H$ | **n$_H$** | **m.** | ***J*[Hz]** |
|---|---|---|---|---|---|---|---|
| **GlcNAc$^A$** | **1** | 88.80 | D | 4.519 | 1 | m | |
| | **2** | 54.63 | D | 3.509 | 1 | m | |
| | **3** | 72.64 | D | 3.509 | 1 | m | |
| | **4** | 78.88 | D | 3.431 | 1 | m | |
| | **5** | 76.74 | D | 3.365 | 1 | ddd | 9.8, 5.1, 2.1 |
| | **6** | 60.25 | T | 3.631 | 1 | dd | 12.3, 2.1 |
| | | | | 3.437 | 1 | dd | 12.3, 5.1 |
| | **2-CO** | 175.06 | S | - | 0 | | |
| | **2-Ac** | 22.37 | Q | 1.812 | 3 | s | |
| **GalNAc$^B$** | **1** | 101.95 | D | 4.286 | 1 | d | 8.4 |
| | **2** | 52.80 | D | 3.692 | 1 | dd | 10.9, 8.4 |
| | **3** | 70.93 | D | 3.512 | 1 | m | |
| | **4** | 67.87 | D | 3.704 | 1 | dd | 3.0, 0.7 |
| | **5** | 75.59 | D | 3.485 | 1 | ddd | 8.2, 4.2, 0.7 |
| | **6** | 61.22 | T | 3.562 | 1 | dd | 11.8, 8.2 |
| | | | | 3.528 | 1 | dd | 11.8, 4.2 |
| | **2-CO** | 175.04 | S | - | 0 | | |
| | **2-Ac** | 22.44 | Q | 1.830 | 3 | s | |

a

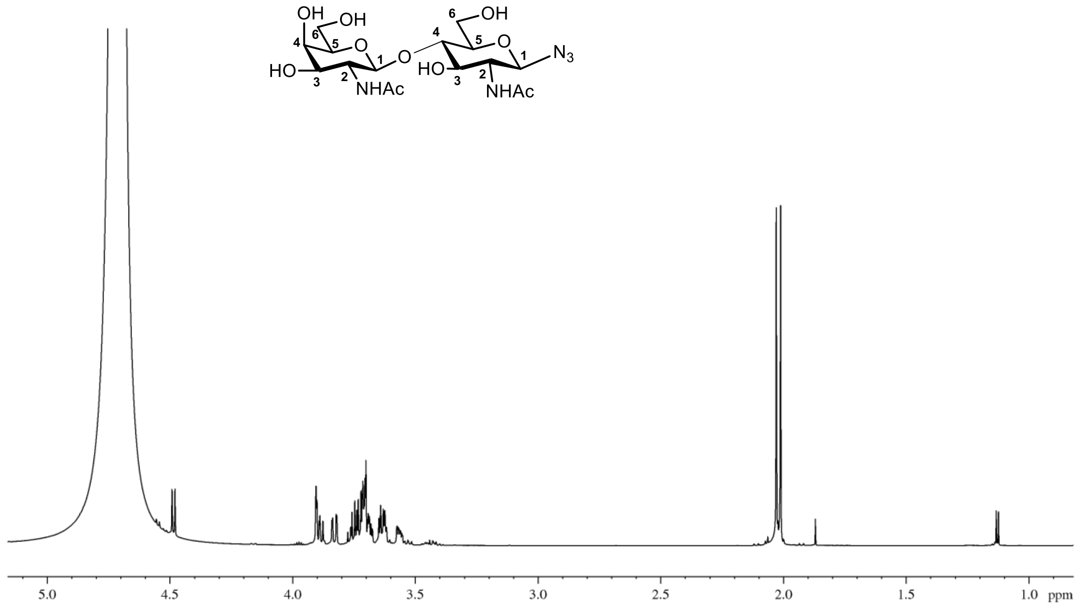


b

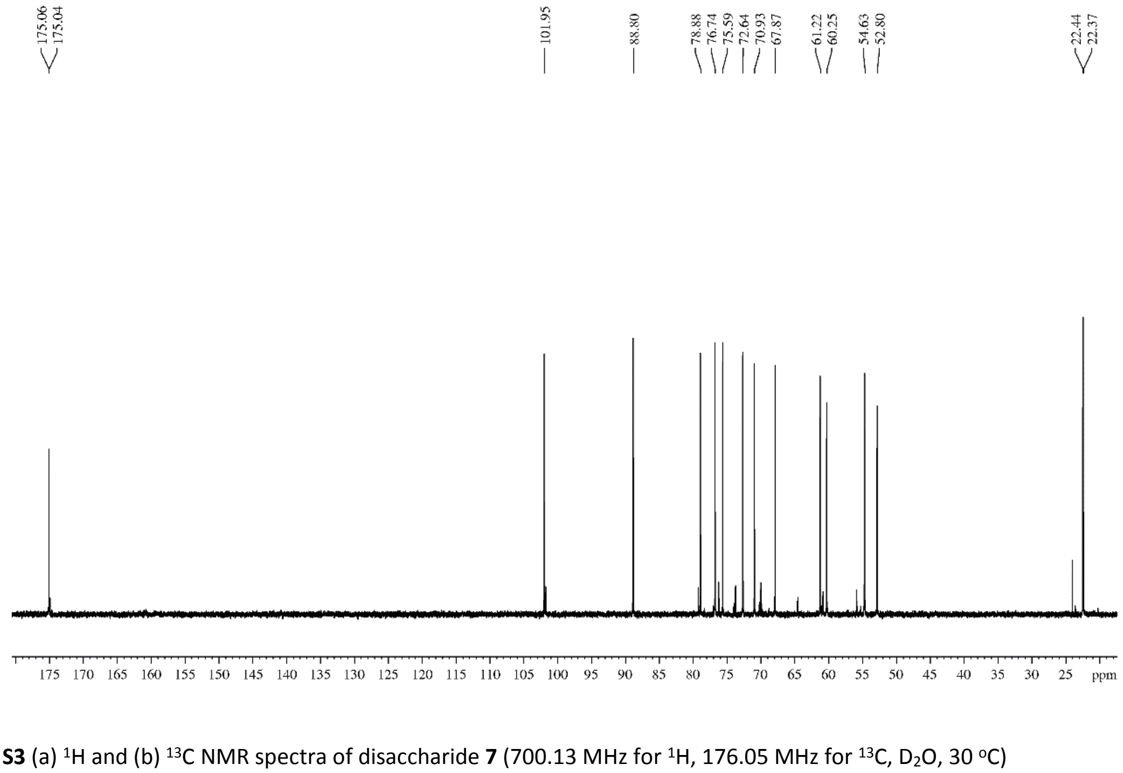


**Fig. S3** (a) $^{1}H$ and (b) $^{13}C$ NMR spectra of disaccharide **7** (700.13 MHz for $^{1}H$, 176.05 MHz for $^{13}C$, $D_2O$, 30 $^{o}C$)

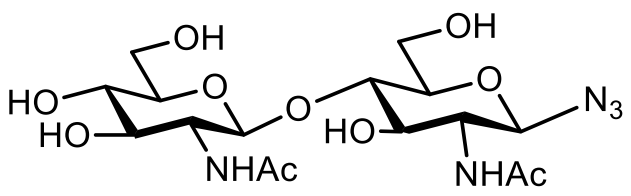


**Fig S4** Structure of 2-acetamido-2-deoxy-β-D-glucopyranosyl-(1→4)-2-acetamido-2-deoxy-β-D-glucopyranosyl azide. The measured $^{1}H$ and $^{13}C$ NMR data and MS data were in a good agreement with the previously published results [1].

## 1.2. MS spectra

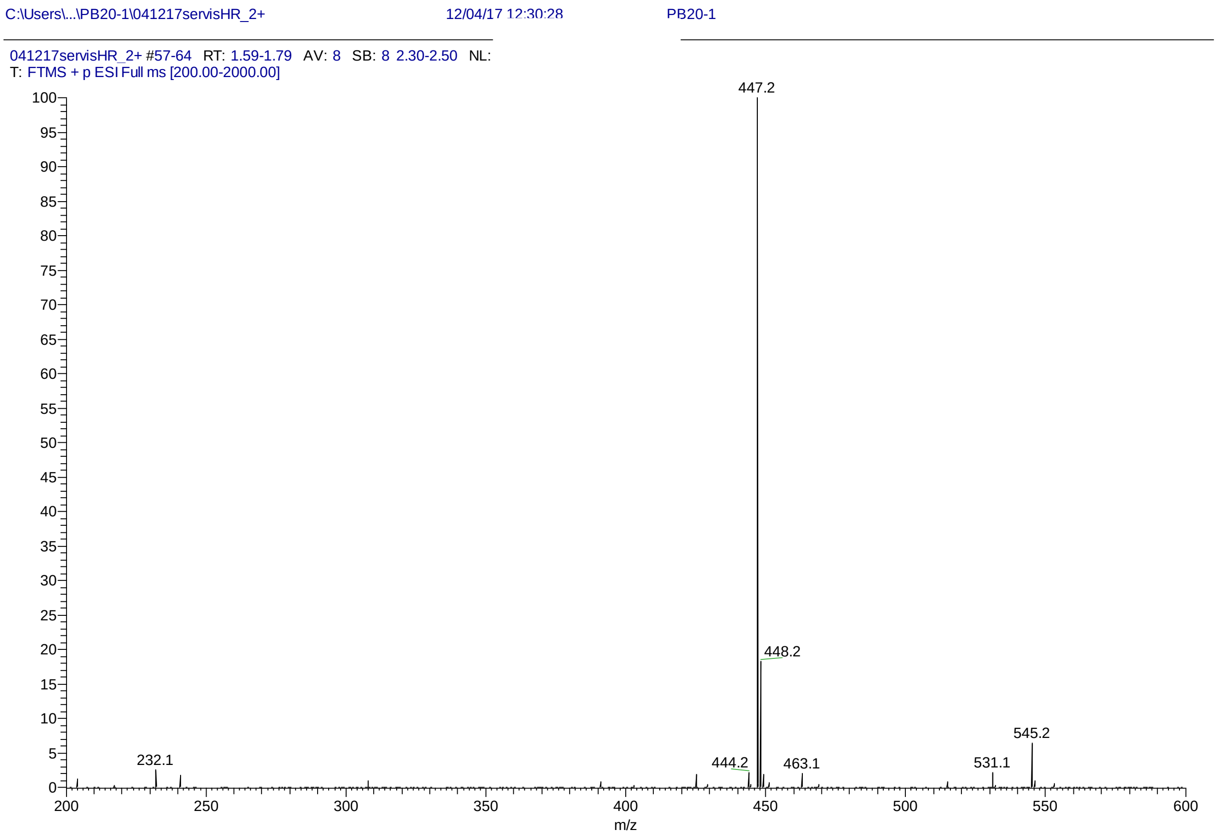


**Fig. S5** MS spectrum (ESI+) of compound **5** ([M + K]+, *m/z* 461.3; [M + Na]+, *m/z* 447.2)

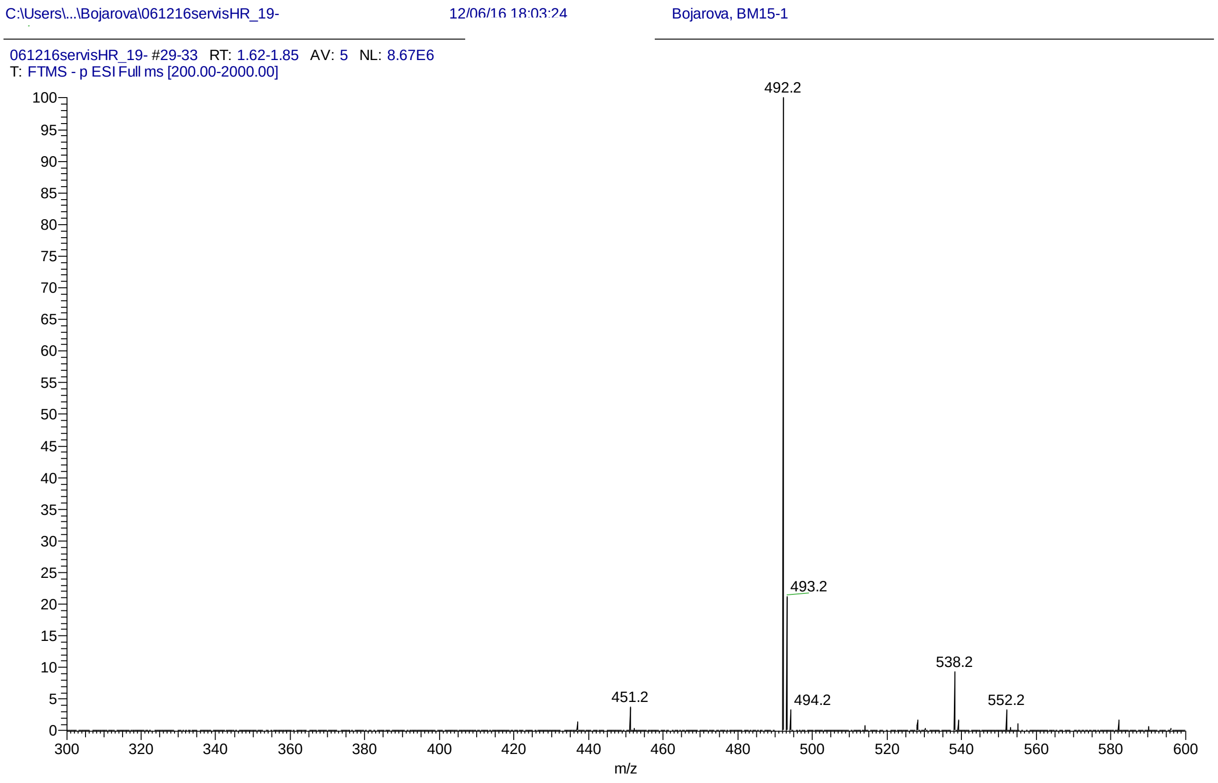


**Fig S6** MS spectrum (ESI$^-$) of compound **6** ($[M-H]^-$, *m/z* 492.2; $[M+HCOO]^-$, *m/z* 538.2; $[M+CH_3COO]^-$, *m/z* 552.2)

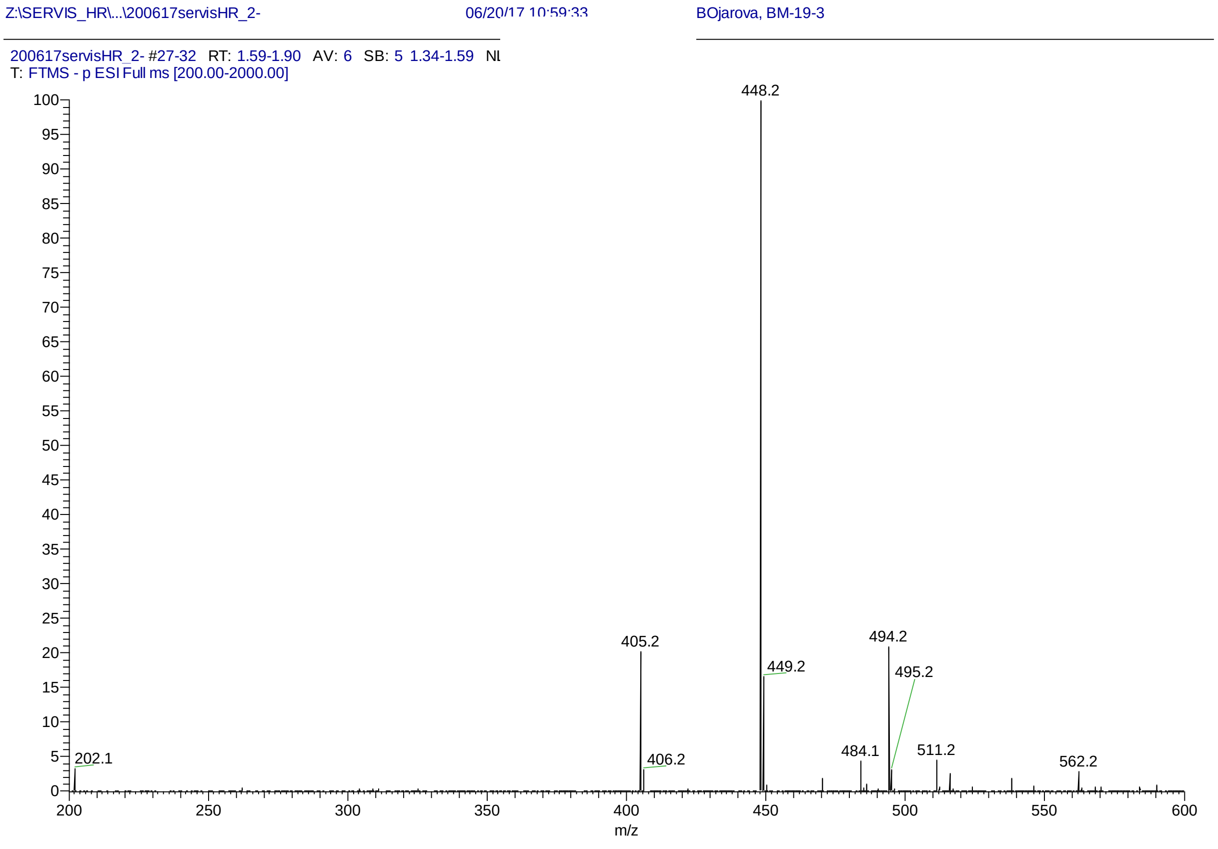


**Fig. S7** MS spectrum (ESI-) of compound **7** ($[M - H]^-$, *m/z* 448.2; $[M + HCOO]^-$, *m/z* 494.2; $[M – H - HN_3]^-$, *m/z* 405.2)

1.3. HPLC chromatograms

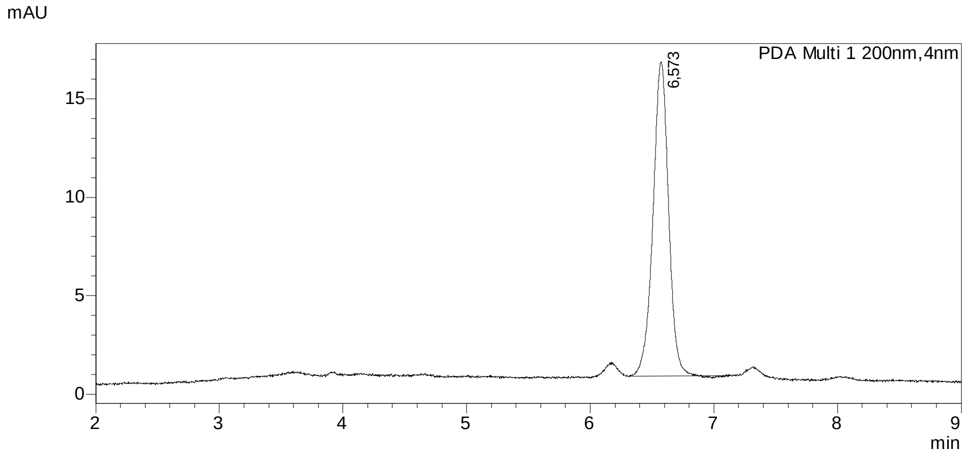


**Fig S8** HPLC chromatogram of 2-acetamido-2-deoxy-β-D-galactopyranosyl-(1→4)-2-acetamido-2-deoxy-D-glucopyranose (LacdiNAc; **5**).

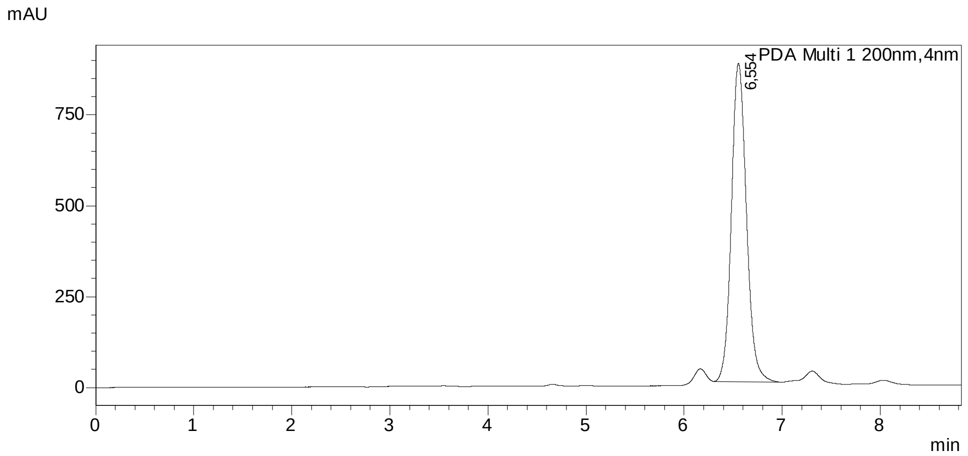


**Fig S9** HPLC chromatogram of 2-azidoethyl 2-acetamido-2-deoxy-β-D-galactopyranosyl-(1→4)-2-acetamido-2-deoxy-β-D-glucopyranoside (**6**).

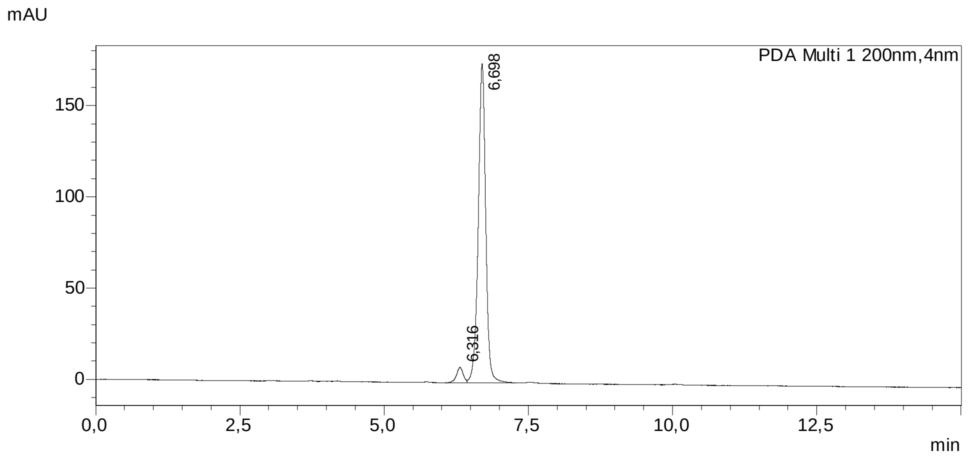


**Fig S10** HPLC chromatogram of 2-acetamido-2-deoxy-β-D-galactopyranosyl-(1→4)-2-acetamido-2-deoxy-β-D-glucopyranosyl azide (6.698 min).

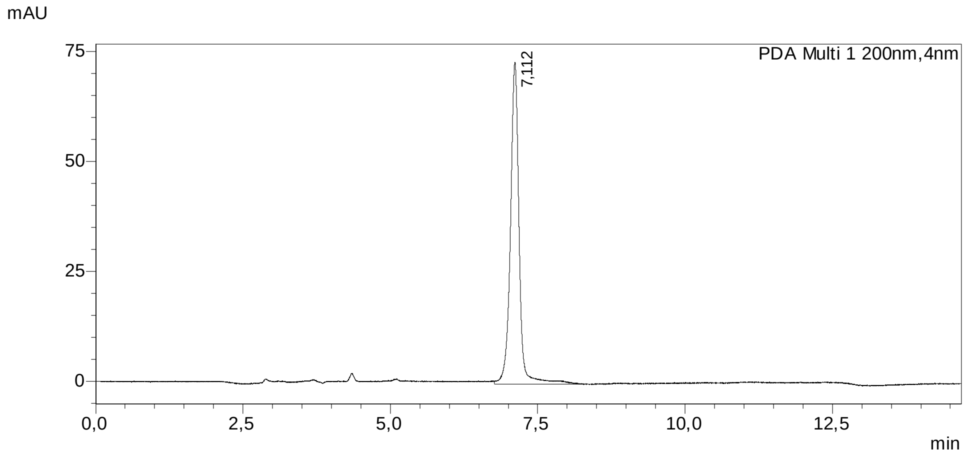


**Fig S11** HPLC chromatogram of 2-acetamido-2-deoxy-β-D-glucopyranosyl-(1→4)-2-acetamido-2-deoxy-β-D-glucopyranosyl azide (7.112 min).

# 2. Glyconanomaterials – synthesis and characterization

## 2.1. Synthesis of polymer precursors

The syntheses are shown in Scheme 2 of the main text.

**General procedure for the synthesis of polymer precursors 10-12**. The polymer precursors bearing thiazolidine-2-thione (TT) groups poly(HPMA-*co*-MA-AP-TT) (**10**-**12**) were prepared by reversible addition–fragmentation chain transfer (RAFT) polymerization of *N*-(2-hydroxypropyl)methacrylamide (HPMA; **8**) and 3-(3-methacrylamidopropanoyl) thiazolidine-2-thione (MA-AP-TT; **9**) using the chain transfer agent 2-cyanopropan-2-yl dithiobenzoate (CTA) and the initiator 2,2′-azobisisobutyronitrile (AIBN), followed by dithiobenzoate end group removal. The reaction conditions were adapted from our previous work [2]. The molar ratio of monomer/CTA/AIBN was 350/2/1 (copolymers **10**, **11**) or 450/2/1 (copolymer **12**) and the molar ratio of reactants **8**/**9** was 9/1 (copolymer **10**), 8/2 (copolymer **11**) or 7/3 (copolymer **12**).

**Example of the synthesis of polymer precursor 10.** HPMA (1.5 g, 10.5 mmol) was dissolved in *tert*-butyl alcohol (10.35 mL) and mixed with a solution of MA-AP-TT (300.7 mg, 1.16 mmol), AIBN (9.32 mg, 33.2 μmol), and CTA (14.7 mg, 67 μmol) in dimethylsulfoxide (DMSO) (2.60 mL, resulting in 0.9 M solution of monomers). The reaction mixture was poured into a glass ampoule, bubbled with argon and sealed. After 16 h in a thermostat controlled water bath at 70°C, the ampoule was cooled, and the reaction mixture was poured into an excess of a mixture of dry acetone and diethyl ether (2/1; 300 mL). After centrifugation at 7800 rpm for 5 min, followed by decantation of the precipitate, the copolymer was purified by precipitation from methanol (13 mL) into the mixture of dry acetone and diethyl ether (2/1; 300 mL). The copolymer was filtered off and

dried under vacuum (1.29 g, 72 % yield). For the removal of the dithiobenzoate end group, the intermediate copolymer and AIBN (193 mg) were dissolved in *N*,*N*-dimethylacetamide (DMA) (9 mL), poured into a glass ampoule, bubbled with argon and sealed. After 2 h in a thermostat controlled water bath at 80 °C, the ampoule was cooled, and the copolymer was isolated by precipitation into the mixture of dry acetone and diethyl ether (2/1; 200 mL). The precipitate was filtered off and purified by precipitation from methanol (9 mL) into the same mixture. The copolymer was filtered off and dried under vacuum to yield the title compound **10** (1.09 g).

**General procedure for the synthesis of polymer precursors 13-15**. The polymer precursors bearing propargyl groups poly(HPMA-*co*-MA-AP-propargyl) (**13**-**15**) were synthesized by aminolysis of TT groups of respective copolymers poly(HPMA-*co*-MA-AP-TT) (**10**-**12**) with propargylamine in DMA using *N*,*N*-diisopropylethylamine (DIPEA) as a base under the reaction conditions described previously [2].

**Example of the synthesis of polymer precursor 13.** Propargylamine (60 µL, 0.93 mmol) and DIPEA (162 µL, 0.93 mmol) were added to the stirred solution of copolymer **10** (1.09 g, 0.62 mmol of TT groups) dissolved in DMA (7 mL). The reaction was carried out at 24 °C for 40 min. The polymer was purified using Sephadex LH-20 column with methanol elution and UV detection. The polymer-containing fraction was concentrated *in vacuo* to 6 mL, and the polymer precursor was isolated by precipitation into an excess of the mixture of dry acetone and diethyl ether (2/1; 200 mL) followed by filtration and drying under vacuum to yield the title compound **13** (0.70 g).

**Synthesis of polymer precursor 16**. The polymer precursor bearing dibenzocyclooctyne (DBCO) groups poly(HPMA-*co*-MA-AP-DBCO) (**16**) was synthesized by aminolysis of TT groups of the copolymer poly(HPMA-*co*-MA-AP-TT) (10) with DBCO-amine in DMA using DIPEA as a base by the similar procedure as described above. A solution of DBCO-amine (13.9 mg, 50 µmol) in DMA (300 µl) was added to the stirred solution of the copolymer **10** (150 mg, 86 µmol of TT groups) in DMA (3 mL). The reaction was carried out at 24 °C for 40 min. The unreacted TT groups were quenched with 1-aminopropan-2-ol (7.4 µl, 97 µmol) and the polymer was purified using Sephadex LH-20 column with methanol elution and UV detection. The polymer-containing fraction was concentrated *in vacuo* to 3 mL, and the polymer precursor was isolated by precipitation into an excess of acetone and diethyl ether (2/1; 100 mL), followed by filtration and drying under vacuum to yield the title compound **16** (127 mg).

**General procedure for the synthesis of polymer precursors 17 and 18.** Polymer precursors with different contents of ethynylphenyl groups (poly(HPMA-*co*-MA-AP-ethynylphenyl); **17** and **18**) were synthesized *via* aminolysis of TT groups of respective copolymers poly(HPMA-*co*-MA-AP-TT) (**10**, **11**) with (4-ethynylphenyl)methanamine in DMA using DIPEA as a base by the similar procedure as described above.

**Example of the synthesis of polymer precursor 17**. A solution of copolymer **10** (150 mg, 86 µmol of TT groups) in DMA (2 mL) was added to the stirred solution of (4-ethynylphenyl)methanamine hydrochloride (21.8 mg, 0.13 mmol) and DIPEA (23 µl, 0.13 mmol) in DMA (1 mL). The reaction was carried out at 24 °C for 20 h. The polymer was purified using Sephadex LH-20 column with methanol elution and UV detection. The polymer-containing fraction was concentrated *in vacuo* to 2 mL, and the polymer precursor was isolated by precipitation into an excess of dry acetone and diethyl ether (2:1; 100 mL) followed by filtration and drying *in vacuo* to yield the title compound **17** (89 mg).

**Synthesis of polymer precursor 20.** The polymer precursor poly(HPMA-*co*-APMA) (**20**) was prepared by RAFT polymerization of HPMA (**8**) and *N*-(3-*tert*-butoxycarbonylaminopropyl)methacrylamide (APMA-Boc; **19**), followed by the removal of dithiobenzoate end group and Boc group. HPMA (0.5 mg, 3.49 mmol) was dissolved in deionized water (2.74 mL) and mixed with a solution of APMA-Boc (142 mg, 0.62 mmol), 4-cyano-4-(thiobenzoylthio)pentanoic acid (6.9 mg, 24 µmol), and 4,4´-azobis(4-cyanopentanoic acid) (ACVA; 3.3 mg, 12 µmol) in 1,4-dioxane (1.37 mL, resulting in 1 M solution of monomers). The reaction mixture was poured into a glass ampoule, bubbled with argon and sealed. After 7 h in a thermostat controlled water bath at 70 °C, the ampoule was cooled, and the reaction mixture was poured into an excess of acetone (100 mL). After centrifugation at 7800 rpm for 5 min, followed by decantation of the precipitate, the copolymer was purified by precipitation from methanol (5 mL) into the mixture of acetone and diethyl ether (2/1; 100 mL). The copolymer was filtered off and dried under vacuum (0.36 g, 57 % yield). The removal of the dithiobenzoate end group was done as described above for polymer **10**. Afterwards, the intermediate copolymer was dissolved in the mixture TFA/ triisopropylsilane/ water 95/ 2.5/ 2.5 (vol. %) at a concentration of 10 wt. %, precipitated into excess of diethyl ether followed by filtration. The product was dissolved in water and pH of the solution was adjusted with NaOH to ca 7-8. The copolymer was isolated by gel filtration (Sephadex G25, eluent water) and freeze-dried (0.27 g).

**Synthesis of compound 22**. 3,5-Diethynylbenzoyl thiazolidine-2-thione (**22**) was prepared by the reaction of 2-thiazoline-2-thiol and 3,5-diethynylbenzoic acid using carbodiimide. 3,5-Diethynylbenzoic acid (**21;** 150 mg, 0.88 mmol) and 1-ethyl-3-(3-dimethylaminopropyl)carbodiimide hydrochloride (EDC.HCl; 404 mg, 2.11 mmol) were dissolved in dichlormethane (8 mL) and cooled to -18 °C. 2-Thiazoline-2-thiol (210 mg, 1.76 mmol) and a catalytic amount of 4-dimethylaminopyridine were dissolved in dichloromethane (5 mL) and cooled to -18 °C. Both solutions were combined and

the reaction mixture was cooled for another 2 h at -18 °C and then left overnight at 4 °C. The dichloromethane solution was washed with brine (3 × 100 mL) and with water (1 × 100 mL) and the organic layer was dried over anhydrous $MgSO_4$. After $MgSO_4$ was filtered off, the dichloromethane solution was concentrated under reduced pressure and the title compound **22** (185 mg, 77 % yield) was isolated by crystallization from ethyl acetate. The purity of the product was checked by a Shimadzu HPLC system equipped with a C18 reversed-phase Chromolith Performance RP-18e column, showing a single peak with a retention time of 3.1 min. TLC: ethyl acetate/hexane (1/1, v/v), $R_f$ = 0.75.

**Synthesis of polymer precursor 23**. The polymer precursor poly(HPMA-*co*-APMA-diethynylphenyl) (**23**) was prepared by the aminolytic reaction of precursor **20** with 3,5-diethynylbenzoyl thiazolidine-2-thione (**22**) in DMA using DIPEA as a base by the similar procedure as described above. A solution of the copolymer **20** (150 mg, 89 μmol of $NH_2$ groups) in DMA (2 mL) was added to the stirred solution of (4-ethynylphenyl)methanamine hydrochloride (21.83 mg, 130 μmol) and DIPEA (23 μl, 130 μmol) in DMA (1 mL). The reaction was carried out at 24 °C for 20 h. The polymer was purified using Sephadex LH-20 column with methanol elution and UV detection. The polymer-containing fraction was concentrated in vacuum to 2 mL, and the polymer precursor was isolated by precipitation into an excess of dry acetone and diethyl ether (2/1; 100 mL) followed by filtration and drying *in vacuo* to yield the title compound **23** (89 mg).

### 2.2. Synthesis of glyconanomaterials

The syntheses are shown in Scheme 2 and all structures are shown in Scheme 3 of the main text.

**General procedure for the synthesis of glycomaterials 24-32.** Glycomaterials **24**-**32** were prepared by the reaction of propargyl groups in polymer precursors **13**-**15** with the respective azido-functionalized carbohydrates **6** or **7** by $Cu^+$-catalyzed azide–alkyne cycloaddition (CuAAC) in water. The first example represents the synthesis of all conjugates except for **28** and **32** containing the highest disaccharide content. Due to their limited solubility in methanol, the purification was modified; the explicit procedure is described below from compound **28**.

**Example of the synthesis of glycomaterial 25.** A solution of $CuSO_4{\cdot}5H_2O$ (2.0 mg, 16 μmol) in water (23 μL) was added to a mixture of the copolymer **10** (25 mg, 17.4 μmol of propargyl groups), sodium ascorbate (1.60 mg, 16.2 μmol), and disaccharide **6** (8.0 mg, 16.2 μmol) dissolved in water (230 μL). The reaction mixture was bubbled with argon before and after the addition of $CuSO_4{\cdot}5H_2O$. The reaction was carried out under magnetic stirring at 24 °C for 1 h. Afterwards, PBS buffer containing EDTA disodium salt (5 wt. %; 1 mL) was added and the title compound was purified by gel filtration using Sephadex G-25 (PD10 column). The polymer fraction was freeze-dried and diluted in methanol (2 mL). An excess of 8-quinolinol was added and after 20 min the sample was purified from the remaining copper using Sephadex LH-20 column with methanol as a mobile phase. The polymer fraction was concentrated under vacuum, diluted in water and freeze-dried to yield the title compound (18.4 mg).

**Example of the synthesis of glycomaterial 28**. A solution of $CuSO_4{\cdot}5H_2O$ (5.3 mg, 42.2 μmol) in water (60 μL) was added to a mixture of copolymer **12** (25 mg, 46.7 μmol of propargyl groups), sodium ascorbate (4.2 mg, 42.2 μmol), and disaccharide **6** (20.82 mg, 42.2 μmol) dissolved in water (190 μL). The reaction mixture was bubbled with argon before and after the addition of $CuSO_4{\cdot}5H_2O$. The reaction was carried out under magnetic stirring at 24 °C for 1 h. After this, PBS buffer with EDTA disodium salt (5 wt. %, 1 mL) was added and the polymer was purified by gel filtration by Sephadex G-25 using water as a mobile phase. The polymer fraction was freeze-dried and the sample was diluted in 2 mL of DMSO. The 8-quinolinol (42 μmol) was added and after 20 minutes the reaction mixture was poured into an excess of ethyl acetate (100 mL). After centrifugation at 7800 rpm during 5 min, the precipitate was filtered and purified by precipitation from DMSO solution (1 mL) into ethyl acetate (50 mL). The title compound was filtered off, diluted in water and freeze-dried (27 mg).

**General procedure for the synthesis of glycomaterials 33-34.** Glycomaterials **33**-**34** were prepared by the reaction of DBCO groups in polymer precursor **16** with the respective azido-functionalized carbohydrates **6** or **7** by Cu(I)-free azide–alkyne cycloaddition in methanol.

**Example of the synthesis of glycomaterial 33**. A solution of disaccharide **6** (4.7 mg, 9.6 μmol) in methanol (350 μL) was added into the stirred solution of the copolymer **16** (14 mg, 4.6 μmol of DBCO) in methanol (400 μL). The reaction mixture was bubbled with argon and carried out under magnetic stirring at 24 °C for 20 h. The polymer product was purified using Sephadex LH-20 column with methanol elution. The polymer fraction was concentrated under vacuum to approximately 1 mL and isolated by precipitation into an excess of ethyl acetate (50 mL) followed by filtration and drying under vacuum (27.5 mg).

**General procedure for the synthesis of glycomaterials 35-37.** Glycomaterials **35**-**37** were prepared by the reaction of ethynylphenyl groups in polymer precursors **17** or **18** with the respective azido-functionalized carbohydrates **6** or **7** by CuAAC in the mixture of water and DMF.

**Example of the synthesis of glycomaterial 35**. A solution of $CuSO_4{\cdot}5H_2O$ (2 mg, 16.0 μmol) in water (20 μL) was added to a mixture of copolymer **17** (25 mg, 14.3 μmol of ethynylphenyl groups), sodium ascorbate (1.6 mg, 16.0 μmol), and disaccharide **7** (7.2 mg, 16.0 μmol) dissolved in the mixture of water and DMF (1/1, v/v; 270 μL). The reaction mixture was

bubbled with argon before and after the addition of $CuSO_4{\cdot}5H_2O$. The reaction was carried out under magnetic stirring at 24 °C for 20 h. After this, PBS buffer with EDTA disodium salt (5 wt. %, 1 mL) was added and the polymer was purified by gel filtration by Sephadex G-25 using water as a mobile phase. The polymer fraction was freeze-dried and the sample was diluted in methanol (1 mL). An excess of 8-quinolinol was added and after 20 min the sample was purified from the remaining copper using Sephadex LH-20 column with methanol elution. The solvent was evaporated under vacuum, the title compound was diluted in water and freeze-dried (16 mg).

**General procedure for the synthesis of glycomaterials 38-39.** Glycomaterials **38**-**39** were prepared by the reaction of diethynylphenyl groups in polymer precursor **23** with the excess of the respective azido-functionalized carbohydrates **6** or **7** by CuAAC in the mixture of water and DMF.

**Example of the synthesis of glycomaterial 39**. A solution of $CuSO_4{\cdot}5H_2O$ (3.74 mg, 15 μmol) in water (45 μL) was added to a mixture of copolymer **23** (19 mg, 12 μmol of ethynyl groups), sodium ascorbate (3 mg, 15 μmol), and disaccharide **7** (6.74 mg, 15 μmol) dissolved in a mixture of water and DMF (1/1, v/v; 210 μL). The reaction mixture was bubbled with argon before and after the addition of $CuSO_4{\cdot}5H_2O$. The reaction was carried out under magnetic stirring at 24 °C for 20 h. After this, PBS buffer with EDTA disodium salt (5 wt. %; 1 mL) was added and the polymer was purified by gel filtration using Sephadex G-25 and water as the eluent and the polymer fraction was freeze-dried. The powder was diluted in methanol (2 mL) and an excess of 8-quinolinol was added. After 20 min the title compound was purified from the remaining copper using Sephadex LH-20 column with methanol elution. The solvent was evaporated under vacuum, the title compound **39** was diluted in water and freeze-dried (14 mg).

## 2.3. NMR spectra

The structures of glycomaterials and their precursors were analyzed using NMR Bruker Avance III 600 spectrometer operating at 600.2 MHz using DMSO-$d_6$ or $D_2O$ as solvents. A representative sample of each group of compounds was chosen to illustrate. In the case of compound **22**, the sample was analyzed using $^1$H-NMR (300 MHz).

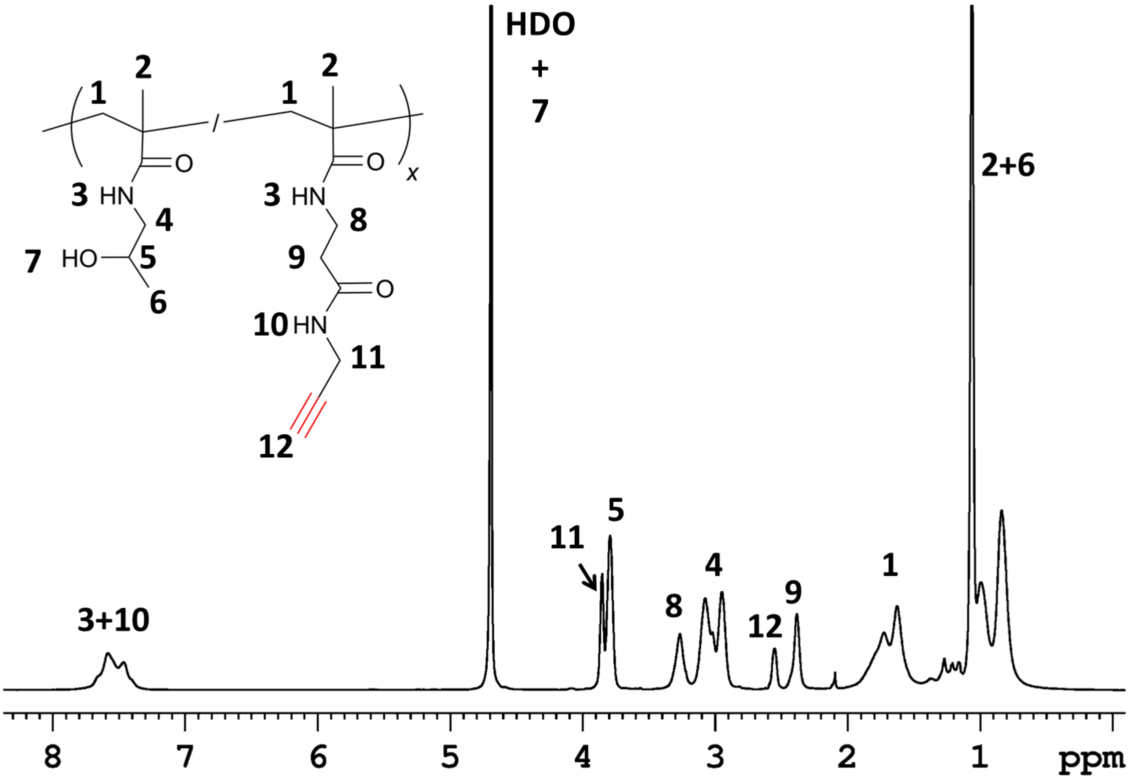


**Fig S12** $^1$H NMR spectrum of copolymer precursor **14** in $D_2O$.

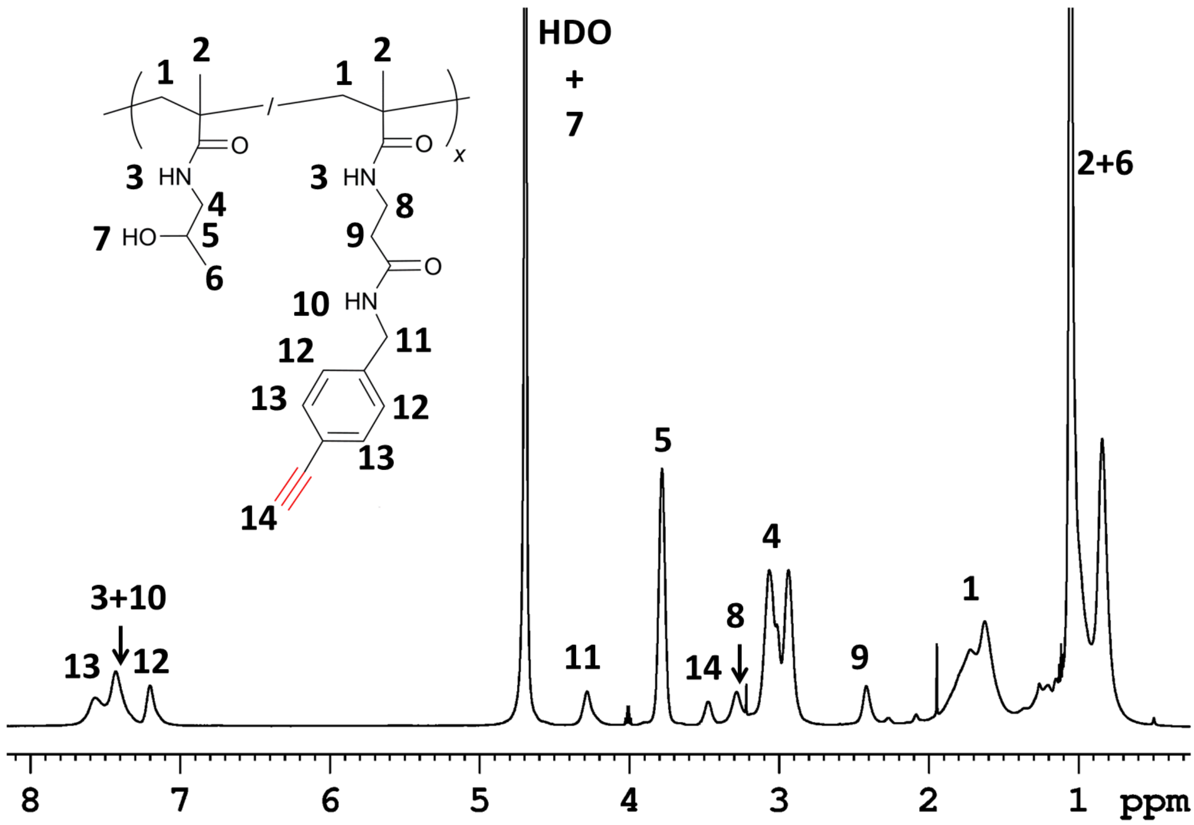


**Fig S13** $^{1}$H NMR spectrum of copolymer precursor **17** (containing 9.9 mol. % ethynylphenyl linker) in $D_2O$.

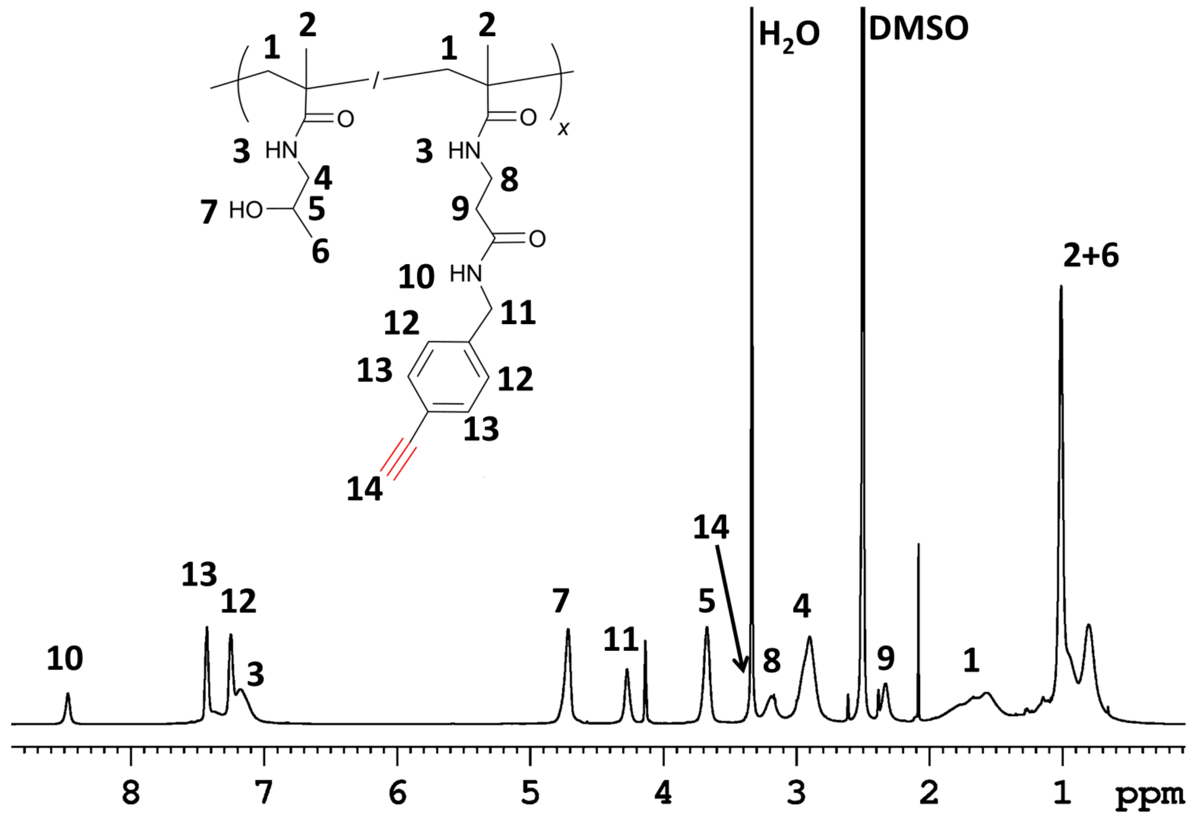


**Fig S14** $^{1}$H NMR spectrum of copolymer precursor **18** (containing 20.2 mol. % ethynylphenyl linker) in DMSO-$d_6$.

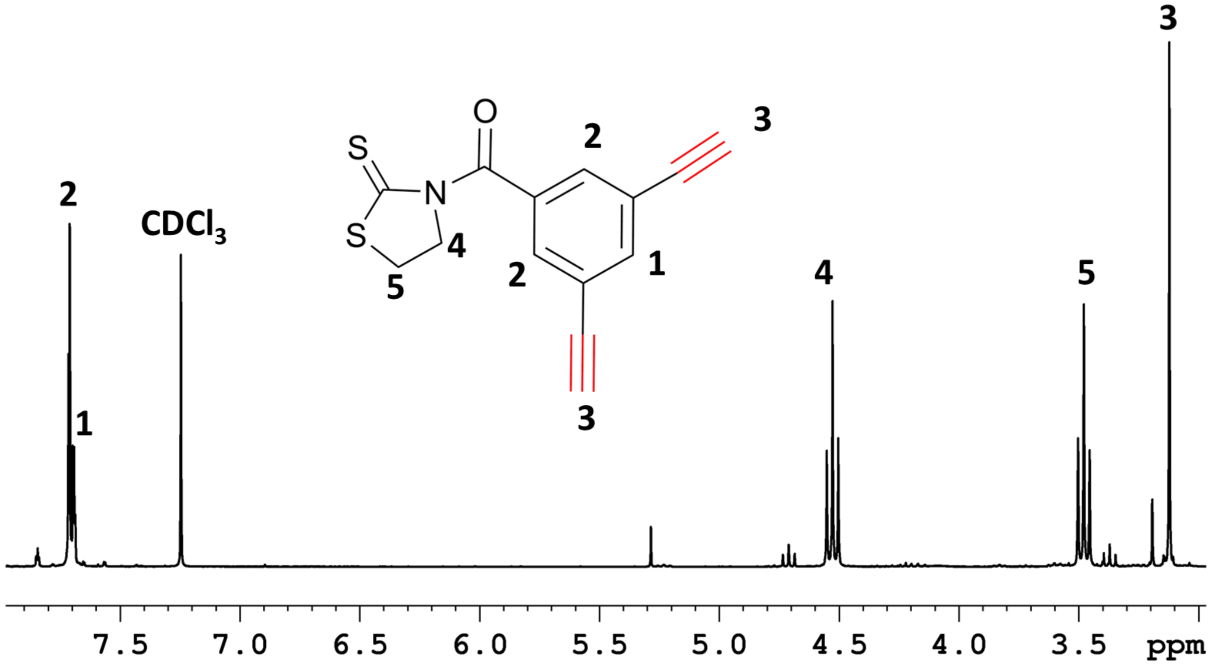


**Fig S15** $^1$H NMR spectrum of compound **22** in $CDCl_3$.

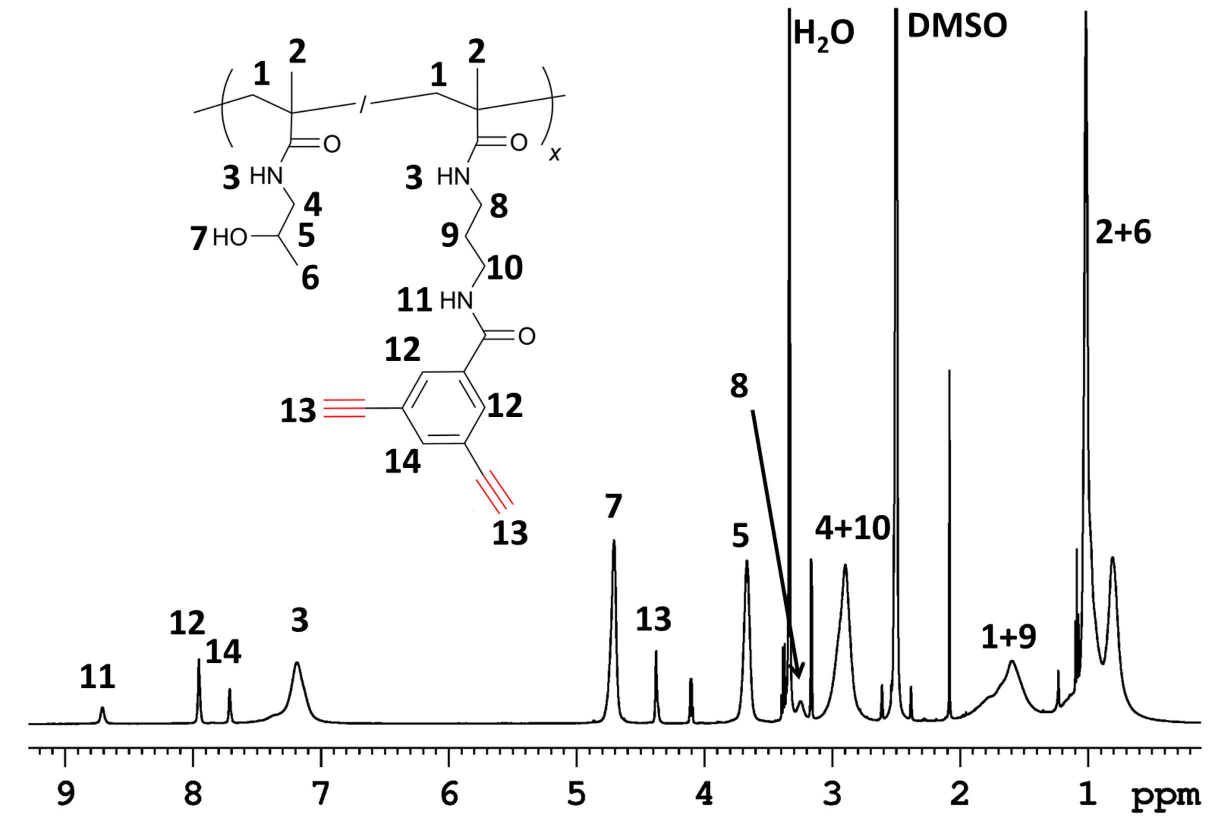


**Fig S16** $^1$H NMR spectrum of copolymer precursor **23** in DMSO-$d_6$.

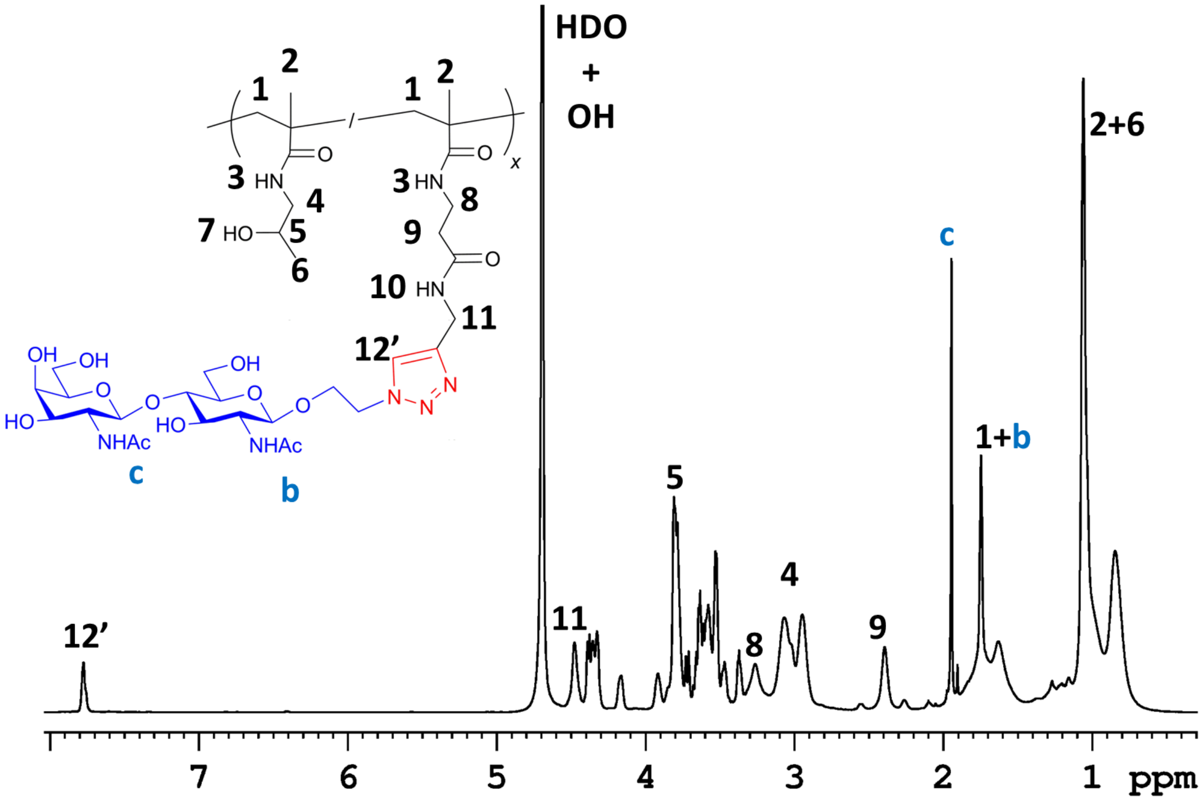


**Fig S17** $^{1}$H NMR spectrum of glycopolymer **27** in $D_2O$.

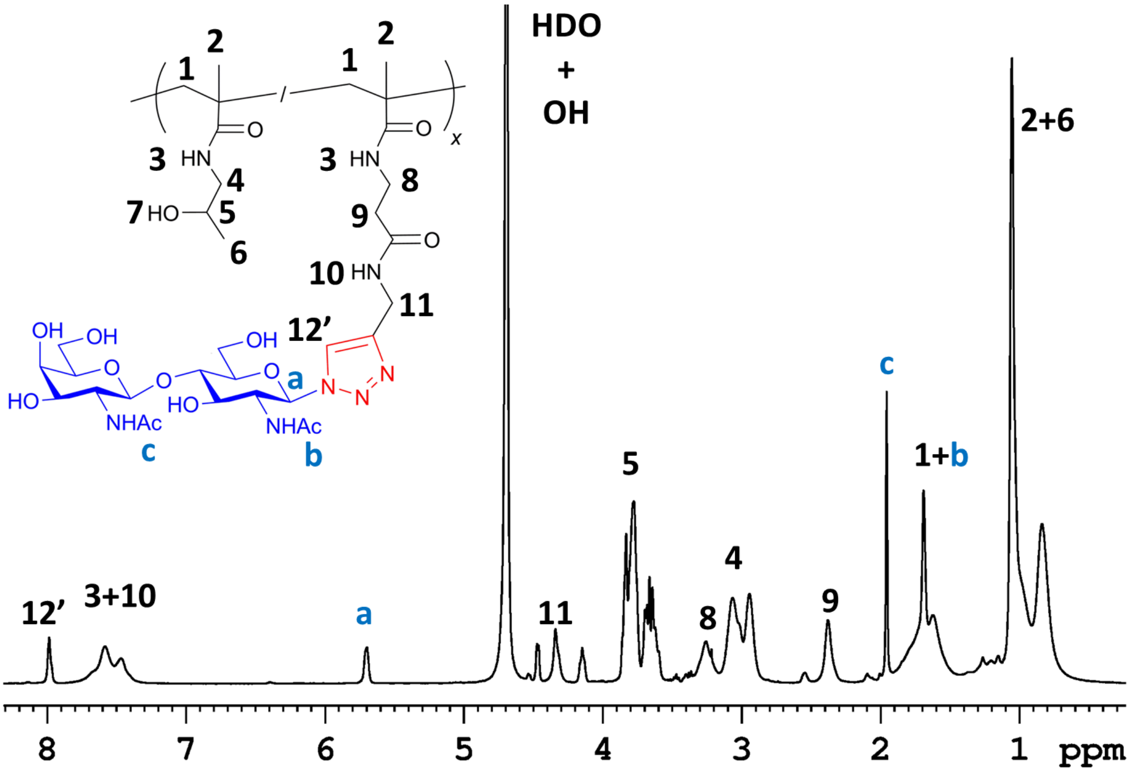


**Fig S18** $^{1}$H NMR spectrum of glycopolymer **31** in $D_2O$.

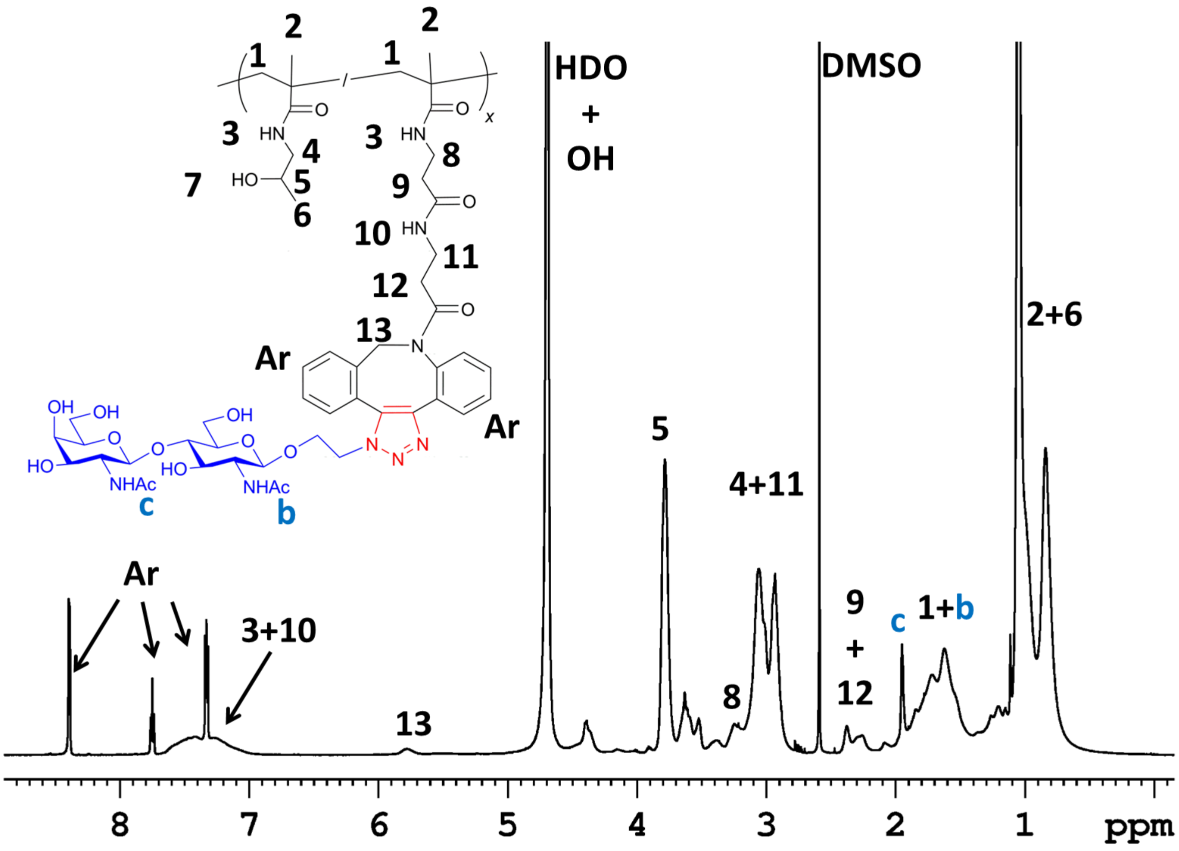


**Fig S19** $^1$H NMR spectrum of glycopolymer **33** in $D_2O$.

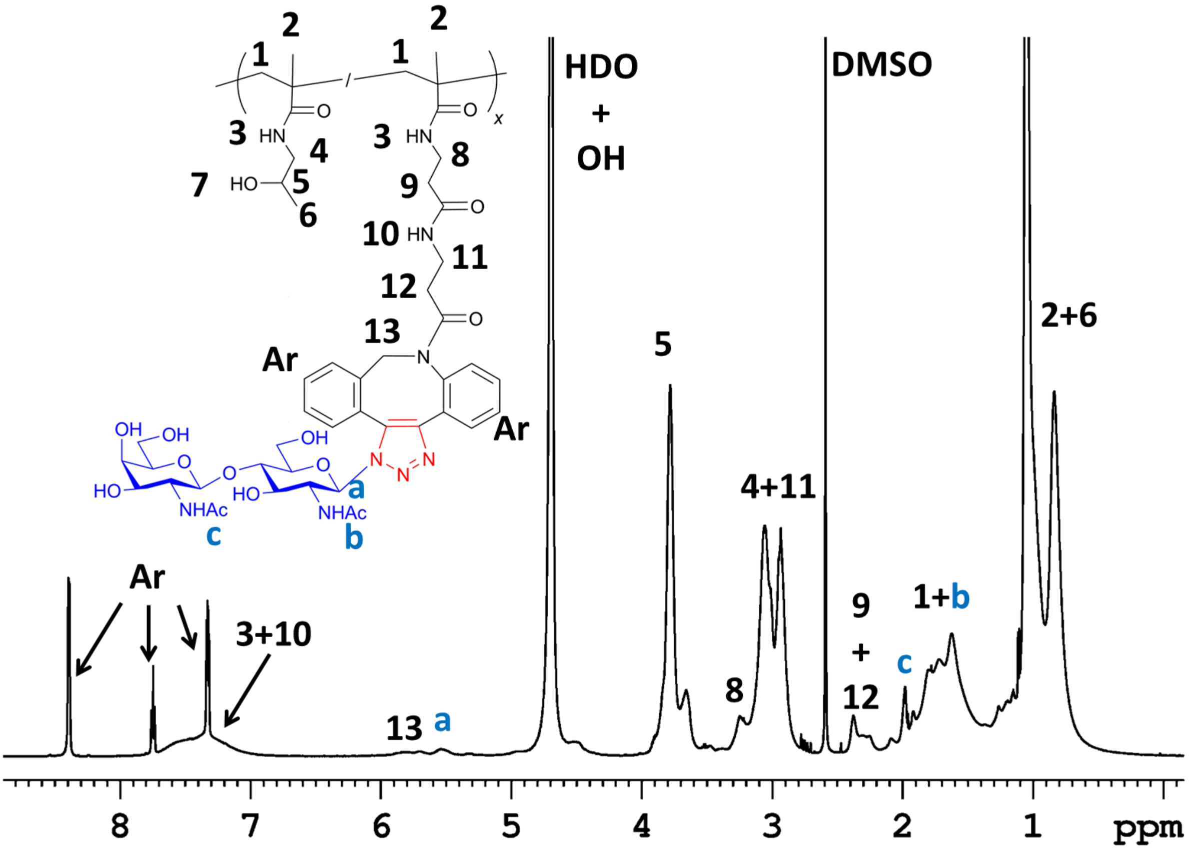


**Fig S20** $^1$H NMR spectrum of glycopolymer **34** in $D_2O$.

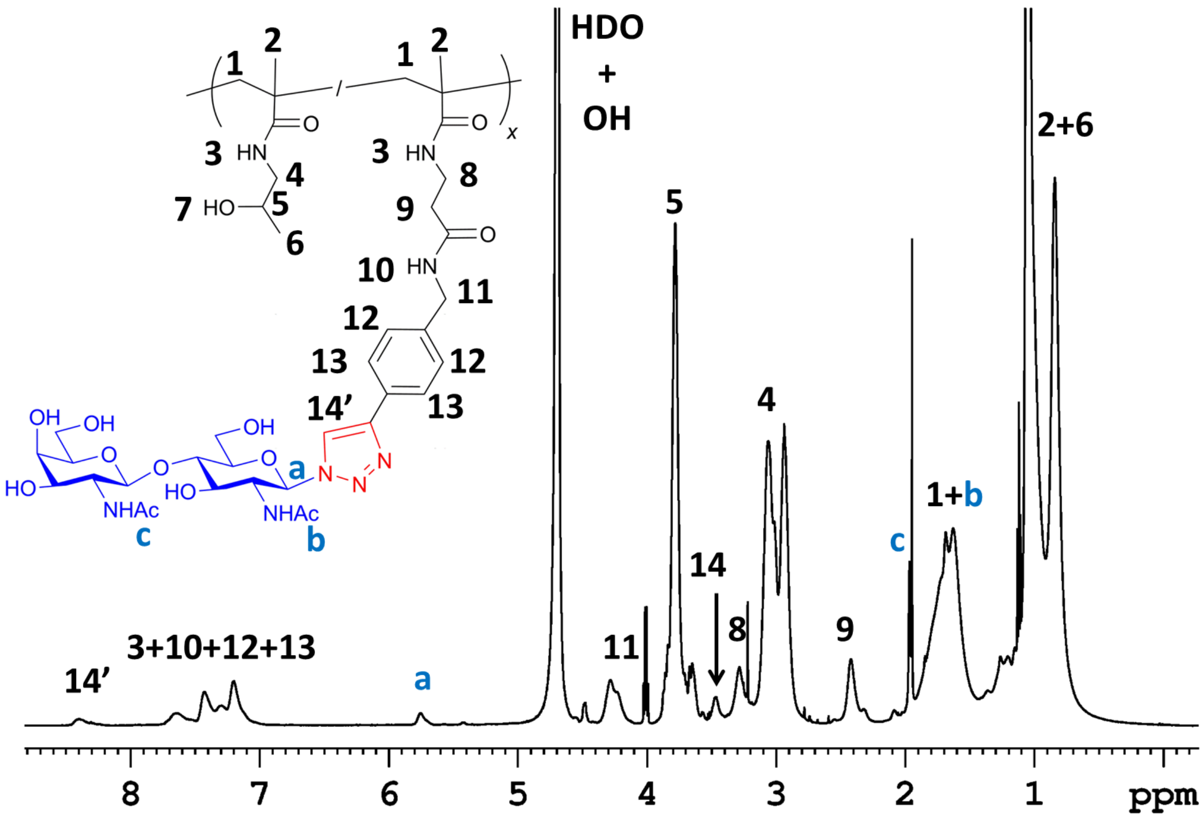


**Fig S21** $^{1}H$ NMR spectrum of glycopolymer **35** in $D_2O$. Hydrogen no. 14 is related to the remaining ethynyl groups.

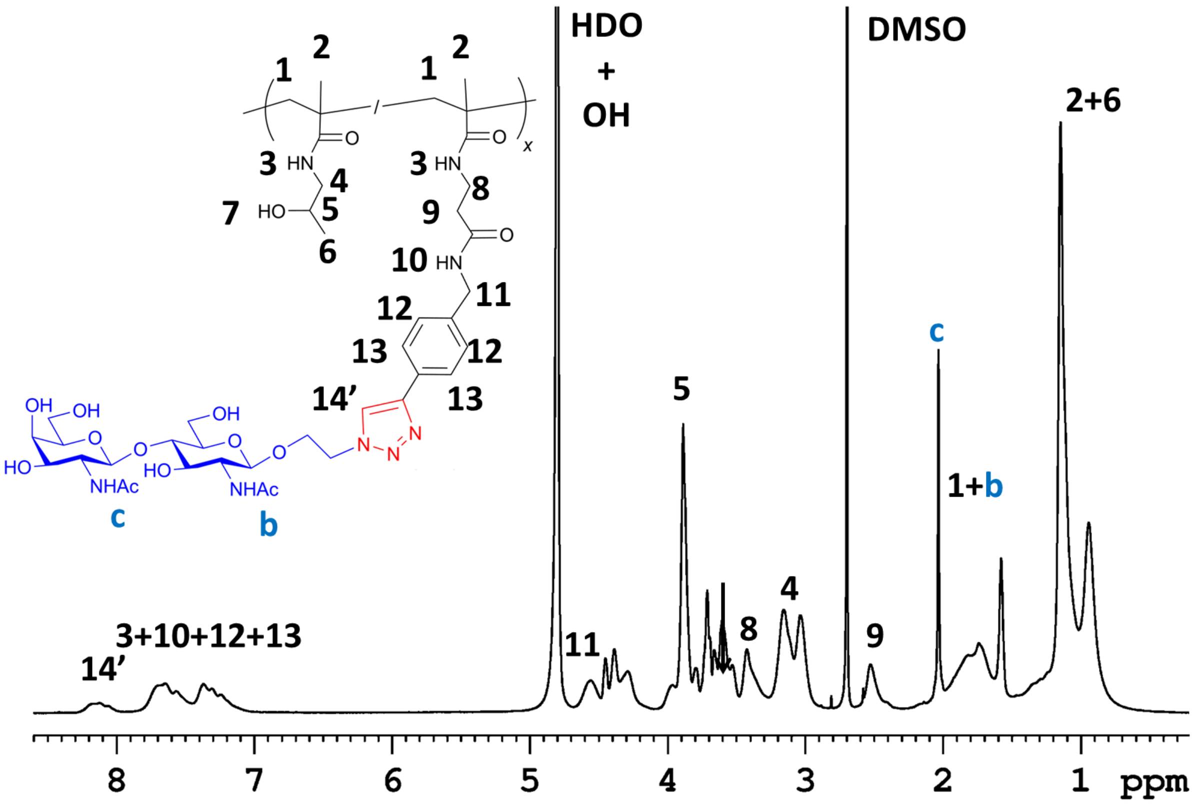


**Fig S22** $^{1}H$ NMR spectrum of glycopolymer **37** in $D_2O$.

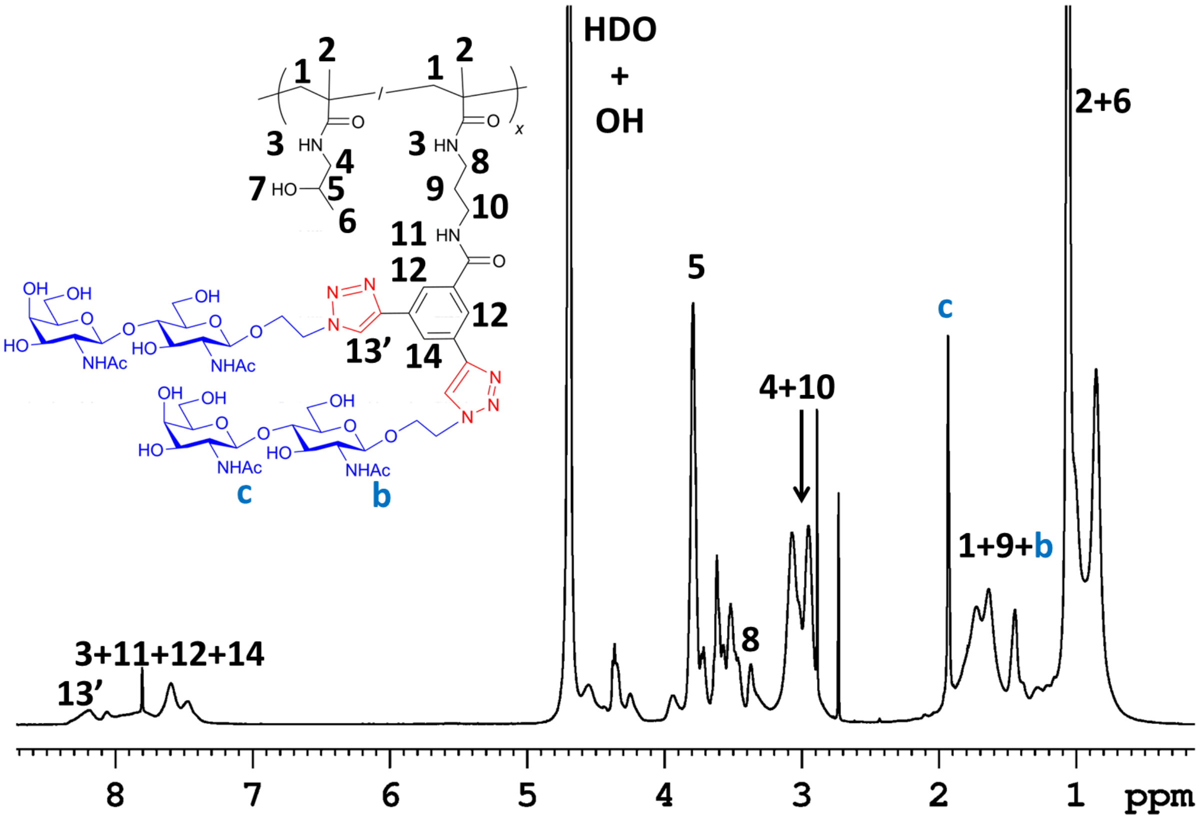


**Fig S23** $^{1}H$ NMR spectrum of glycopolymer **38** in $D_2O$.

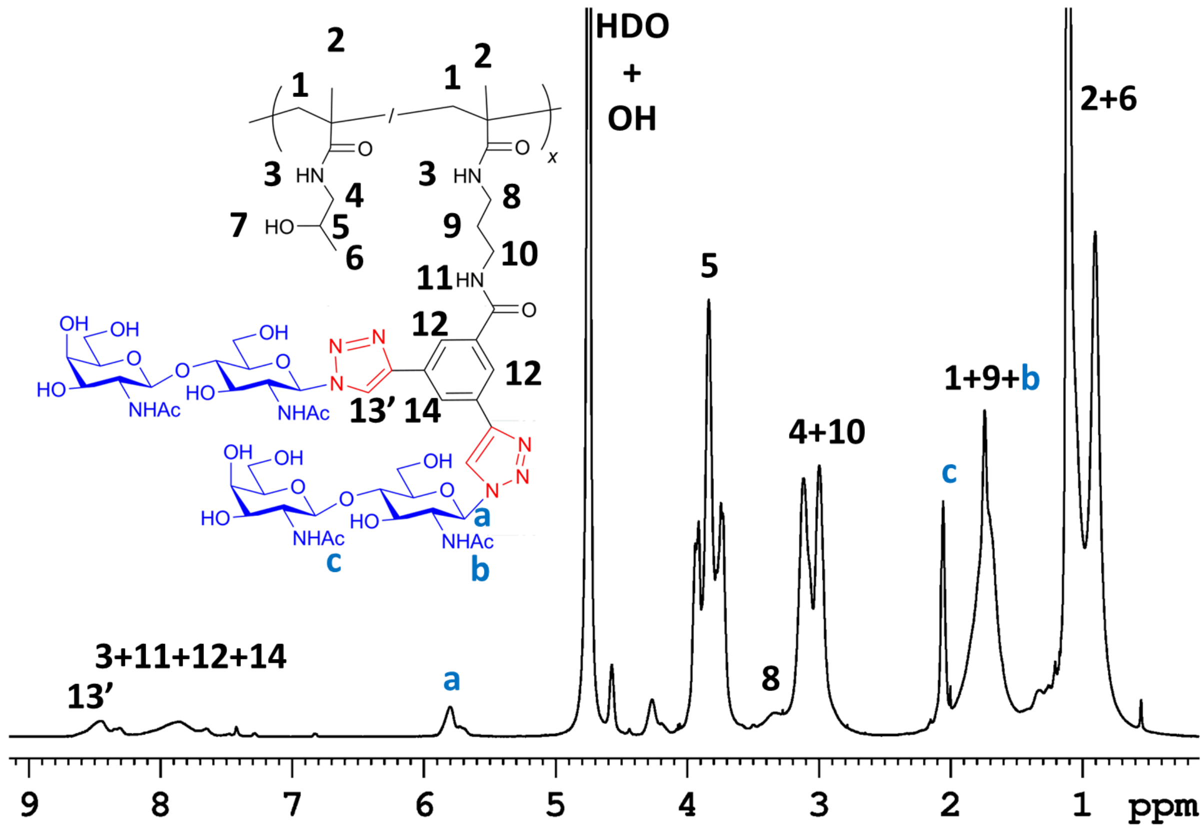


**Fig S24** $^{1}H$ NMR spectrum of glycopolymer **39** in $D_2O$.

## 2.4. Hydrodynamic radii

**Table S4** Hydrodynamic radius of copolymers **13**, **29**-**32**, **38** and **39**

| Compound | Sugar motif with spacer | Content of saccharide (mol. %) | $R_H$ (nm)[a] |
|---|---|---|---|
| **13** | - | 0 | 3.5 ± 0.1 |
| **29** | LacdiNAc-*N*-triazole | 2.7 | 3.4 ± 0.1 |
| **30** | LacdiNAc-*N*-triazole | 8.1 | 3.7 ± 0.2 |
| **31** | LacdiNAc-*N*-triazole | 12.0 | 4.6 ± 0.1 |
| **32** | LacdiNAc-*N*-triazole | 24.8 | 3.3 ± 0.2 |
| **38** | (LacdiNAc-*O*Et-triazole)$_2$Ph | 12.3 | 4.4 ± 0.3 |
| **39** | (LacdiNAc-*N*-triazole)$_2$Ph | 14.0 | 4.8 ± 0.3 |

[a] Hydrodynamic radii ($R_H$) were determined in water by dynamic light scattering at a concentration of 5 mg mL$^{-1}$. They were not extrapolated to zero concentration.

**References**


[1] P. Fialová, A. T. Carmona, I. Robina, R. Ettrich, P. Sedmera, V. Přikrylová, L. Petrásková-Hušáková and V. Křen, Glycosyl azides - Novel substrates for enzymatic transglycosylations, *Tetrahedron Lett*. 2005, **46**, 8715-8718.

[2] P. Bojarová, P. Chytil, B. Mikulová, L. Bumba, R. Konefał, H. Pelantová, J. Krejzová, K. Slámová, L. Petrásková, L. Kotrchová, J. Cvačka, T. Etrych and V. Křen, Glycan-decorated HPMA copolymers as high-affinity lectin ligands, *Polym. Chem*. 2017, **8**, 2647-2658.
